# Biochemical profile and biofunctional effects of *Crithmum maritimum* L. from three harvests under different N:P:K ratios

**DOI:** 10.3389/fnut.2026.1833407

**Published:** 2026-05-13

**Authors:** Beatriz H. Paschoalinotto, Nikolaos Polyzos, Tânia C. S. P. Pires, Adriana K. Molina, Filipa Mandim, Miguel A. Prieto, Maria Inês Dias, Lillian Barros, Spyridon A. Petropoulos

**Affiliations:** 1 Centro de Investigação de Montanha (CIMO), LA SusTEC, Instituto Politécnico de Bragança, Campus de Santa Apolónia, Bragança, Portugal; 2 Instituto de Agroecoloxía e Alimentación (IAA), Universidade de Vigo, Nutrition and Food Group (NuFoG), Campus Auga, Ourense, Spain; 3 Laboratory of Vegetable Production, Department of Agriculture, Crop Production and Rural Environment, University of Thessaly, Volos, Greece

**Keywords:** antimicrobial properties, antioxidant activity, nutritional value, phenolic compounds, rock samphire, sea fennel, wild edible species

## Abstract

**Introduction:**

In the present study, we investigated the effects of different fertilization regimes on the biochemical profile and bioactive properties of *Crithmum maritimum* over three consecutive growing periods.

**Methodology:**

The applied fertilization regimes included six treatments that differed in the amounts of N:P:K (mg/L), namely, 100:100:100 (CM_111_), 200:100:100 (CM_211_), 200:200:200 (CM_222_), 300:100:100 (CM_311_), 300:200:200 (CM_322_), and 300:300:300 (CM_333_) mg/L of N:P:K, and the control treatment (CM_0_; no fertilizers added). The fresh biomass yield of the aboveground part was evaluated for three consecutive growing periods (2021–2023), while biochemical and functional properties assessment included the evaluation of proximate composition, lipophilic (free sugars and organic acids) and hydrophilic compounds (tocopherols and fatty acids) content, phenolic compounds profile, antioxidant activity (thiobarbituric acid reactive substances (TBARS) and oxidative hemolysis inhibition assay (OxHLIA)), and antimicrobial and anti-inflammatory properties.

**Results and discussion:**

High nutrient inputs (CM_333_) significantly increased the fresh biomass yield in the 1st harvest, whereas a lower amount of nutrients led to higher productivity in the successive harvests (CM_322_ and CM_222_ for the 2nd and 3rd harvest, respectively). This study demonstrated that high nutrient inputs in *C. maritimum* significantly enhanced the levels of fat, protein, fiber, and energy in the 2nd and 3rd harvest, while the control treatment (CM_0_) recorded the highest fat and energy content in the 1st harvest. Moreover, low to moderate inputs favored higher ash and carbohydrate content. Glucose remained the dominant sugar, with total sugar levels peaking under high fertilization in later harvests, whereas in the 1st harvest the CM_0_ recorded the highest content of fructose, trehalose and total sugars content. Fertilization impacted organic acids in a varied manner: low inputs favored quinic and succinic acids, whereas higher nutrient levels increased oxalic and malic acid content. *α*-Τocopherol was the sole tocopherol detected, peaking under the CM322 treatment. Regarding fatty acids, high nutrient inputs promoted unsaturated fats and reduced palmitic acid, with the second harvest providing the most nutritious profile and optimal PUFA (polyunsaturated fatty acids)/SFA (saturated fatty acids) and omega-6/omega-3 ratios, while the control treatment had the highest SFA content in the 2nd and 3rd harvest. The second harvest also yielded the highest total phenol content under high-input regimes, whereas lower inputs peaked in the first and third harvests. Antioxidant performance fluctuated by fertilization regime and harvesting, with the second harvest generally showing the highest overall capacity. *Crithmum maritimum* extracts exhibited limited antimicrobial efficacy compared to standard controls and no activity against fungi, and they showed moderate inhibition against specific pathogens, such as *Staphylococcus aureus*, *Escherichia coli*, and methicillin-resistant *S. aureus* (MRSA), depending on the treatment. Finally, the extracts demonstrated no significant anti-inflammatory effects across any tested fertilization regimes.

**Conclusion:**

In conclusion, moderate to high nutrient inputs seem to beneficially affect fresh yield in *C. maritimum*, whereas targeted nutrient inputs may regulate the chemical profile and improve the quality of the edible leaves.

## 1 Introduction

The accretive global demand for food, in conjunction with climate change, progressive soil salinization, and scarcity of irrigation water, has engendered considerable challenges for modern conventional agricultural systems (1–3). Therefore, there is a growing demand for alternative and less exploited crops capable of combining agronomic resilience with high nutritional value to ensure food security and safety in the years to come (4). Conventional crops, such as rice, maize, onions, carrots, wheat, and lettuce, are classified as glycophytes, meaning that the absorption of nutrients and water by these species is impaired by excess salt (1). Conversely, halophytes exhibit a high level of tolerance to abiotic stresses, enabling growth and reproduction under extreme environmental conditions, including diverse levels of salinity and drought (3, 5). Despite comprising only 1–2% of terrestrial plant species, these plants have emerged as promising candidates for sustainable agriculture, owing to their ability to synthesize and accumulate bioactive secondary metabolites in stress response, which helps the plants sustain growth and development under arduous conditions (1, 5–8).

Among edible halophytes, *Crithmum maritimum* L. (Apiaceae), known as sea fennel or rock samphire, has attracted particular attention owing to its wide global distribution, high ecological adaptation, and exceptional nutritional and phytochemical properties (9, 10). The plant has a long rhizomatous root and a branched woody stem; its leaves are elongated, thick, and succulent, attached to the stem by a small petiole, whereas during the reproductive period, erect flowering stalks are formed with several umbels of characteristic yellow-green flowers (7, 9).

From a sensory perspective, sea fennel is characterized by a salty and slightly bitter taste, frequently compared to that of fennel and celery, which is generally accepted in culinary contexts (3, 5, 7). Traditionally, in Mediterranean countries, including Greece, Croatia, Cyprus, Spain, and Tunisia, the aerial parts (leaves, flowers, and stalks) have been consumed mainly in the form of preserves in vinegar or olive oil, in salads, soups, sauces, and as a condiment for fish and vegetables (6, 7, 9–11). The essential oils of the aerial parts also contain significant amounts of bioactive compounds and exert potent antioxidant activities in ethnopharmacology and traditional folk medicine (12–15). In addition to its culinary use, previous studies have shown that the species is also a significant source of fiber, minerals, essential fatty acids (such as omega 3 and 6), vitamins, phenolic acids, flavonoids, and aromatic compounds, with bioactive properties, namely antioxidant, antimicrobial, and anti-inflammatory, which support its promising potential as a functional food and nutraceutical ingredient (3, 9, 10, 16). From an ecological perspective, wild populations of *C. maritimum* play an ecologically significant role in coastal ecosystems, contributing to the stabilization of dunes and cliffs through their root systems, while also acting as pioneer species in coastal restoration processes due to their high tolerance to salinity, drought, and wind exposure (9, 10).

Despite being a traditionally used wild edible plant and a species of considerable contemporary interest, sea fennel remains underutilized in European countries, including those in the Mediterranean basin. Studies that comprehensively assess agricultural management and metabolic dynamics across successive harvests of *C. maritimum* are scarce. Recent investigations have primarily focused on the effects of different cultivation practices – including salinity, water stress, nutrient deficiency, and biofortification – on proximate composition, phenolic profiles, and plant biomass production from a single harvest of *C. maritimum* (1, 5, 17).

The cultivation of halophytes under controlled conditions is essential to dissociate intrinsic metabolic responses from random environmental variations and to promote the optimization of cultivation practices geared towards improved quality of each species (5, 10). In this context, the use of greenhouses for pot cultivation with the application of nutrient solutions enriched with nitrogen, phosphorus, and potassium (N:P:K) may promote the modulation of primary and secondary metabolites under easily controlled conditions. Nitrogen is directly associated with the synthesis of proteins and phenolic compounds, phosphorus regulates energy and structural processes, while potassium plays a key role in osmotic balance, photoassimilate transport, and enzyme activation (18, 19). In species adapted to extreme environments, variations in the N:P:K ratio can also act as metabolic stressors, promoting physiological adjustments that favor the accumulation of bioactive compounds without severely compromising plant growth (20–22).

So far, there is little information in the literature regarding the cultivation protocols needed for the commercial exploitation of sea fennel, since most of the studies refer to the chemical characterization of the species using material collected form the wild. However, studies that could promote the cultivation of sea fennel for the production of high added value end-products in terms of functional and bioactive compounds not only respond to contemporary market demands but also emerge as a viable strategy for contributing to the conservation of wild biodiversity and the functionality of agroecosystems. Accordingly, the present study aimed to evaluate the impact of fertilization with different N:P:K ratios and successive harvests on the growth, proximate composition, phytochemical and phenolic profiles, and bioactive properties, namely, anti-inflammatory, antimicrobial, and antioxidant activities, of pot-grown *C. maritimum* plants focusing on establishing best practice guides for its successful valorisation, as well as on identifying natural compounds with antimicrobial and functional properties that could replace synthetic ones in the food and pharmaceutical industry.

## 2 Methodology

### 2.1 Plant material and cultivation conditions

The trial was conducted at the experimental farm of the University of Thessaly in Velestino during September 2020 and July 2023, according to the methodology previously described by Polyzos et al. (23) and Paschoalinotto et al. (24). Briefly, seeds of *Crithmum maritimum* L. were sown in seed trays in September 2020, and young seedlings were transplanted into 6 L plastic containers with peat and perlite (1:1, w/w) in January 2021. Physicochemical properties of peat were as follows: bulk density 0.12 g/cm^3^, water holding capacity 218.5%, pH 6.0, electrical conductivity (EC) 0.35 dS/m, organic matter 47.5%, carbon (C) 27.5%, nitrogen (N) 0.14%, C/N 196.8, P 160 mg/L, and K (cmol/kg) 46.03 (25). The pots remained in an unheated glasshouse throughout the winter and were then transferred outdoors for the remainder of the experimental period (April 2021–July 2023). The meteorological data recorded throughout the experimental period are presented in Supplementary Material (Supplementary Tables S1, S2 for the indoors and outdoors conditions, respectively). We assessed seven fertilization treatment that differed in the amounts of N:P:K in nutrient solution, namely, 100:100:100 (CM_111_), 200:100:100 (CM_211_), 200:200:200 (CM_222_), 300:100:100 (CM_311_), 300:200:200 (CM_322_) and 300:300:300 (CM_333_) mg/mL of N:P:K and the control treatment (CM_0_) in which no fertilizers were added. The details of the nutrient solution composition are presented in detail in previous works by our team (23, 24). Each treatment comprised 15 pots (*n* = 15) with one plant per pot, totalling 105 pots, which were laid out according to a Completely Randomized Design (CRD). All pots were fertigated at regular intervals with the same amount of nutrient solution, ranging from 150 to 250 mL, depending on the conditions. The 1st harvest took place in August 2021. After the first harvest, the plants were left to complete their growth cycle (seed ripening and senescence) and were then trimmed at approximately 5 cm above the substrate surface. The same treatments were applied in 2022 and 2023, following the same procedure as in the first year. The 2nd and 3rd harvests were performed in August 2022 and July 2023, respectively. The recorded traits at harvest included the fresh and dry weight (g) of the leaves per plant. After weighing, three pooled fresh samples of leaves from each treatment obtained from five plants each were kept at −80 °C, freeze-dried, ground to a powder, and stored at −80 °C until further analysis (26).

### 2.2 Biochemical profile

The ash, protein (nitrogen × 6.25), lipid, and fibre amounts were evaluated using the Association of Official Analytical Chemists (AOAC) numbers 935.42, 991.02, 989.05, and 985.29, respectively (27). Carbohydrate content was calculated as the remaining amount to complete 100% of the proximate composition. According to the Regulation of the European Union (European Regulation No. 1169/2011, 2011), protein and carbohydrate provide 4 kcal/g, while fat and fibre 9 kcal/g and 2 kcal/g, respectively. Therefore, the energy contribution of the aerial parts of *C. maritimum* was calculated.

#### 2.2.1 Lipophilic constituents

The lipophilic constituents analysed in the dry aerial parts of *C. maritimum* were high-molecular-weight organic acids and tocopherol isoforms, which were determined by gas chromatography (GC) and high-performance liquid chromatography (HPLC), respectively. High-molecular-weight organic acids were obtained by transesterification of the previously obtained lipids (Section 2.2). As outlined by Bertéli et al. (28), lipid extracts were incubated for 12 h with a solution prepared from toluene, sulfuric acid, and methanol. High-molecular-weight organic acids were extracted with diethyl ether, dried over anhydrous sodium sulfate, filtered (0.2 μm nylon), and transferred to vials. GC analysis was performed on a DANI 1000 system with split/splitless injector and flame ionization detector (FID) (260 °C) using a Macherey Nagel column (30 m × 0.32 mm × 0.25 μm df). Oven program: 100 °C for 2 min, ramp 10 °C/min to 140 °C, 3 °C/min to 190 °C, 30 °C/min to 260 °C for 2 min. Hydrogen carrier gas at 4.0 mL/min; injection split 1:50, 1 μL per analysis. They were identified by retention times relative to a 37 standard mixture and expressed as relative percentages. Regarding the tocopherol isoforms, the extraction, identification, and quantification were performed as detailed by Bertéli et al. (28). Briefly, the dried aerial parts of *C. maritimum* were extracted with methanol and hexane, centrifuged, and the hexane layer was collected, dried under a nitrogen flow, and filtered. HPLC was performed on a normal-phase column (Poliamide II, 250 × 4.6 mm, 5 μm, 35 °C) with fluorescence detection (excitation 290 nm, emission 330 nm). The mobile phase was n-hexane:ethyl acetate (70:30 v/v) at 1 mL/min. Tocopherols were quantified relative to the internal standard and expressed as mg/100 g dry weight.

#### 2.2.2 Hydrophilic constituents

Hydrophilic constituents, such as low-molecular-weight organic acids and disaccharides, were measured in the dry aerial parts of *C. maritimum* by chromatographic methods. Briefly, the low molecular weight organic acids, as previously detailed in Bertéli et al. (28), were extracted with metaphosphoric acid for 20 min at room temperature. After filtration, the extracts were analyzed using a Shimadzu UFLC system equipped with a diode array detector (DAD). Separation was achieved on a C18 reversed-phase column (250 × 4.6 mm, 5 μm) at 35 °C under isocratic elution with 3.6 mM sulfuric acid. Detection was carried out at 215 and 245 nm. Disaccharides were determined following the procedure described by Mandim et al. (29) using High-Performance Liquid Chromatography (HPLC) coupled to a refractive index detector (HPLC-RI). Extraction was performed with 80% ethanol, concentrated, defatted with ethyl ether, and redissolved in distilled water. The extracts were injected into HPLC-RI, and separation was performed isocratically with acetonitrile/water (70:30, v/v) at 1 mL/min and 35 °C. Melezitose was used as an internal standard. Compounds were identified and quantified by comparison with commercial standards and calibration curves.

#### 2.2.3 Phenol constituents

To assess the phenolic constituents and biofunctional effects (Section 2.3), the aerial parts of *C. maritimum* underwent extraction at room temperature with continuous agitation, following the methodology outlined by Añibarro-Ortega et al. (30). An 80% (v/v) ethanol solution was used as the extraction solvent. The resulting hydroethanolic extracts were freeze-dried and stored for subsequent analyses.

The hydroethanolic extracts were dissolved in an ethanol:water solution (20:80 v/v) at a concentration of 10 mg/mL. These solutions were analysed using HPLC coupled with a diode array detector and mass spectrometry with an electrospray ionization (ESI) source to identify the phenolic constituents present in *C. maritimum* aerial parts (31). Compound identification was based on their maximum absorption wavelength (λ_max_), retention times (RT), deprotonated ion ([M-H]^−^), and main mass fragments (MS^2^). Quantification was performed using calibration curves prepared with analytical standards, and in cases where standards were unavailable, compounds from chemically similar classes were used.

### 2.3 Biofunctional effects

The anti-inflammatory and antimicrobial (including clinical isolates and American Type Culture Collection (ATCC) bacteria) effects of the hydroethanolic extracts of *C. maritimum* aerial parts were evaluated by inhibiting nitric oxide production and a rapid *p*-iodonitrotetrazolium chloride (INT) colorimetric assay, respectively, as described by Taofiq et al. (32).

Briefly, in the antimicrobial effects, five gram-negative (*Enterobacter cloacae* ATCC 49741, *Salmonella enterocolitis* ATCC 13076, *Eschericia coli* ATCC 25922, *Pseudomonas aeruginosa* ATCC 9027, *Yersinia enterocolitica* ATCC 8610) and three gram positive bacteria (*Listeria monocytogenes* ATCC 19111, *Bacillus cereus* ATCC 11778, *Staphylococcus aureus* ATCC 11632) foodborne were tested, while clinical isolates from hospitalized patients in departments of local health units in Northeast Portugal were also tested, namely, *Morganella morganii*, *Escherichia coli*, *Proteus mirabilis*, *Klebsiella pneumoniae* and *Pseudomonas aeruginosa Enterococcus faecalis*, *Listeria monocytogenes*, methicillin-resistant *Staphylococcus aureus* – MRSA. The incubation between bacteria strain and each extract (10–0.15 mg/mL) took 24 h at 37 °C to keep the exponential phase of growth to define the minimum bactericidal concentration (MBC) and the minimum inhibitory concentration (MIC), representing the lowest concentration able to kill and inhibit the bacteria, respectively. Streptomicin, methicillin, ampicillin, imipenem, and vancomycin were used as positive controls. Further, the antifungal properties were also studied as previously described in our work (33). *Aspergillus fumigatus* (ATCC 204305) and *Aspergillus brasiliensis* (ATTC 16404) were employed. The concentration range was the same as that used for antimicrobial effects to evaluate the MIC or minimum fungicidal concentration (MFC). The fungal spores were exposed to each extract for 72 h at 26 °C. Ketoconazole was used as a positive control.

The antioxidant effect was measured using the thiobarbituric acid reactive substances (TBARS) and oxidative hemolysis inhibition assay (OxHLIA) methods (29, 34). For the TBARS assay, pig brain tissues were homogenized with a Tris–HCl buffer solution and centrifuged. An aliquot of the supernatant was incubated with the extracts, FeSO₄, and ascorbic acid at 37 °C for 1 h. Trichloroacetic acid and thiobarbituric acid solutions were added, and the mixture was heated. After centrifugation, the absorbance of the supernatant was measured. Trolox was used as a positive control. The results were expressed as IC_50_ values, indicating the concentration of the extract providing 50% of the antioxidant activity. In the OxHLIA method, an erythrocyte solution (2.8%, v/v; 200 μL) prepared in phosphate-buffered saline (PBS, pH 7.4) was mixed with 400 μL of either the extract solution (20–4000 μg/mL), Trolox (3.91–4000 μg/mL; positive control), PBS solution (negative control), or distilled water (baseline). After 10 min of incubation at 37 °C with shaking, 200 μL of 2,2′-azobis(2-methylpropionamidine) dihydrochloride (AAPH; 160 mM) was added, and the optical density was measured kinetically at 690 nm in an ELx800 microplate reader (Bio-Tek Instruments, Winooski, VT, USA) until complete hemolysis. The IC_50_ values (μg/mL) were obtained using GraphPad Prism®9 for a Δt of 60 min.

### 2.4 Statistical assessment

Plant growth measurements were performed in 15 plants (*n* = 15) for the fresh weight of leaves per plant and five plants (*n* = 5) for the dry matter content. Chemical analyses were performed in triplicate in three batch samples (*n* = 3) of each treatment. The data were processed using one-way analysis of variance (ANOVA) with the IBM SPSS Statistics 23 software for Windows to identify differences among the fertilization treatments of each harvest for the tested growth, chemical composition and bioactive properties variables. When statistically significant differences were identified, the means were compared using Tukey’s honest significant difference (HSD) test at a significance level of *p* < 0.05. Hierarchical cluster analysis (HCA) was implemented to group the *C. maritimum* samples based on similarities in their chemical profiles. The clustering process used Euclidean distance as the proximity measure and Ward’s method for cluster linkage. To ensure the equal weighting of all variables, the data were standardized prior to analysis. The optimum number of clusters was determined by evaluating the resulting dendrogram and distance graph. For HCA, JMP v. 16.1 (SAS Institute Inc., Cary, NC, USA) statistical software was used.

## 3 Results and discussion

### 3.1 Crop performance

**Figure 1:**
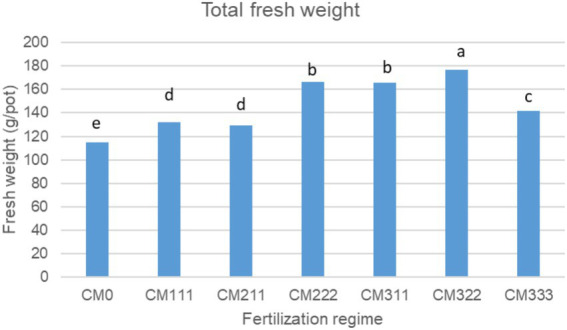
Total fresh weight (g/pot) of *Crithmum maritimum* from three harvests in relation to fertilization regime (*n* = 15).

The effect of the fertilization regime on plant growth was evaluated by assessing the fresh and dry weight of leaves per plant for three consecutive harvests (Table 1). Our results show that during the 1st growing period, the application of the highest amounts of nutrients (CM_333_: 300:300:300 mg/mL N:P:K) resulted in the highest overall fresh biomass production, indicating high nutrient requirements at the beginning of crop establishment. However, nutrient needs gradually decreased in the following years, and the highest fresh leaf weight was recorded for the CM_322_ (300:200:200 mg/mL N:P:K) and CM_222_ (200:200:200 mg/mL N:P:K) in the 2nd and 3rd harvests, respectively. The cumulative fresh weight was also the highest for the CM_322_ treatment (Figure 1). Moreover, the application of low to moderate nutrient inputs (CM_111_ and CM_222_, 100:100:100 mg/mL N:P:K and 200:200:200 mg/mL N:P:K, respectively) and the control treatment (CM_0_: no fertilizers added) resulted in increasing trends of fresh leaf biomass throughout the experimental period (1st to 3rd harvest). In contrast, high inputs (CM_311_, CM_322_, and CM_333_, 300:100:100 mg/mL N:P:K, 300:200:200 mg/mL N:P:K, and 300:300:300 mg/mL N:P:K, respectively) and treatment CM_211_ (200:100:100 mg/mL N:P:K) showed an increasing trend in the first two harvests and a high decrease in the 3rd harvest. Regarding the dry matter content of leaves, the control treatment had the lowest overall content in all the harvests consistently, whereas no significant differences were recorded among most of the treatments in the first two harvests (Table 1).

**Table 1:** Effects of fertilization regimes on fresh and dry weight of leaves/plant (g/pot) and dry weight of leaves (%) of *Crithmum maritimum* (mean ± SD; *n* = 15).

Treatments	Traits	
	Weight of leaves/plant (g/pot)	Dry weight of leaves (%)
*1st harvest*		
CM_0_	42 ± 3^c^	12.3 ± 0.3^b^
CM_111_	34 ± 2^d^	12.9 ± 0.2^b^
CM_211_	43 ± 2^c^	13 ± 1^b^
CM_222_	43 ± 3^c^	13 ± 1^b^
CM_311_	45 ± 2^c^	13 ± 1^b^
CM_322_	51 ± 2^b^	12.4 ± 0.3^b^
CM_333_	59 ± 2^a^	14 ± 1^a^
*2nd harvest*		
CM_0_	51 ± 5^f^	11.8 ± 0.4^c^
CM_111_	53 ± 8^ef^	12.9 ± 0.3^b^
CM_211_	70 ± 9^d^	13 ± 1^a^
CM_222_	59 ± 7^e^	14 ± 1^a^
CM_311_	100 ± 9^b^	14 ± 1^a^
CM_322_	113 ± 11^a^	14 ± 1^a^
CM_333_	80.2 ± 8.1^c^	14 ± 1^a^
*3rd harvest*		
CM_0_	64 ± 5^c^	10.0 ± 0.6^e^
CM_111_	78 ± 8^b^	11.0 ± 0.7^d^
CM_211_	59 ± 5^c^	13.1 ± 0.4^b^
CM_222_	107 ± 16^a^	12.1 ± 0.8^c^
CM_311_	65 ± 9^c^	13.2 ± 0.3^b^
CM_322_	63 ± 6^c^	14.8 ± 0.5^a^
CM_333_	62 ± 8^c^	13.1 ± 0.7^b^

^*^Mean values in the same column and harvest followed by different Latin letters are significantly different at *p* < 0.05 according to Tukey’s honestly significant difference (HSD) test.

According to the literature, the ratio and the amount of macronutrients in nutrient solution may affect the growth of various horticultural species, including wild edible greens, such as *Cichorium spinosum*, *Scolymus hispanicus*, *Portulaca oleraceae* and *Sonchus oleraceus* (18, 23, 24, 35–37). However, given variability in growing conditions and cropping systems (e.g., pot cultivation or soilless hydroponic systems), these effects varied across studies. Moreover, genotypic differences were detected, as the responses of different wild edible greens varied even in studies conducted by our team, despite the same experimental protocol (23, 24).

In particular, Chrysargyris and Tzortzakis (36) evaluated the effect of increasing total (50, 100, 200, and 300 mg/L) and NH_4_^+^-N concentration in the nutrient solution on the growth of hydroponically grown *S. oleraceus* plants and they reported that the fresh and dry aerial biomass increased significantly when total nitrogen exceeded 100 mg/L, with no significant differences between among the 100, 200, and 300 mg/mL treatments. In contrast, the increase of NH_4_^+^-N concentration had the opposite effect on both the fresh and dry weight of the aerial parts of the plant. Similar results were recorded in our study when considering only the final harvest, when plants were fully established, where the balanced solution of moderate amounts of macronutrients (CM_222_) had the best performance in terms of fresh biomass yield. In the first two harvests, higher nutrient inputs were more effective, as *C. maritimum* plants are perennial and exhibit a very slow growth at the beginning of their growth cycle. Paschoalinotto et al. (24) reported similar findings for *S. hispanicus* grown under the same experimental protocol as in our study, with the nutrient solutions of 200:200:200 and 300:200:200 showing the highest fresh weight of leaves, and the control treatment showing the highest leaf dry matter content.

Regarding *C. spinosum*, which is another perennial wild edible species, Polyzos et al. (23) suggested that moderate inputs of N and low inputs of P and K recorded the highest fresh weight of leaves, while the highest leaf dry matter content was also observed for the control treatment. These findings, combined with the results of our study, highlight the importance of the genetic background in the response of plants to nutrient inputs. Therefore, despite the implementation of the same protocol and the fact that the three studied species grow as perennials in their natural habitats, differences in nutrient requirements and species-specific growth habits were reflected in the aerial biomass yield. Petropoulos et al. (38) evaluated the fresh aerial biomass yield of *C. spinosum* plant for two consecutive years, recording a varied yield from harvest to harvest within the same year, as well as on total biomass yield between different years. The same authors also suggested that growing conditions and sowing date may have an effect on fresh biomass yield, especially when multiple harvests are implemented within the same growing period (38). Moreover, Martins-Noguerol et al. (20) evaluated the soil physicochemical properties from samples taken *in situ* from several growing locations, and suggested that plants that grew in less fertile soils were taller and had higher leaf N and P content. In contrast, Castillo et al. (5) reported that nutrient deprivation had no significant effect on the above and below ground biomass of pot-grown *C. maritimum* plants, as salinity stress intensity was more relevant to crop performance compared to nutrient availability.

However, it must be noted that the literature reports regarding the effect of fertilization practices on *C. maritimum* are scarce, while, to the best of our knowledge, the effect of nutrient application on plant growth in a multi-year basis was studied only in the present study. So, our results are not fully comparable with those of other studies in the literature.

### 3.2 Proximate composition

The content of the key components of centesimal composition is presented in Table 2, with a varied response among the implemented fertilization regimes being recorded across the three harvests. In particular, fat content was the highest for the control treatment in the 1st harvest, while nutrient application in high amounts further increased its content in the successive harvests (CM_322_ (300:200:200 mg/mL N:P:K) and CM_333_ (300:300:300 mg/mL N:P:K) for the 2nd and 3rd harvest, respectively). A similar pattern was observed for protein and fibre content, where the treatments with high nutrient inputs (CM_311_ (300:100:100 mg/mL N:P:K), CM_322_ (300:200:200 mg/mL N:P:K), and CM_333_ (300:300:300 mg/mL N:P:K) and CM_322_ (300:200:200 mg/mL N:P:K) for protein and fibre content, respectively) increased their content in *C. maritimum* leaves through the succession of harvests. A contrasting trend was noted for ash content, where low or no inputs were the most beneficial, especially at the last harvest, while carbohydrate content also benefited from treatments with low inputs (e.g., CM_211_ and CM_222_, 200:100:100 mg/mL N:P:K and 200:200:200 mg/mL N:P:K, respectively) throughout the growing period. Finally, energy content followed the trends of fat and protein, which contribute significantly to the overall calorific value of leaves.

**Table 2:** Centesimal composition of *Crithmum maritimum* aerial parts (mean ± SD; *n* = 3).

Centesimal composition (g 100/g dry weight)						Energy (kcal 100 g^−1^ dw)
	Fat	Protein	Ash	Fibre	Carbohydrates	
*1st Harvest*						
CM_0_	3.7 ± 0.1^a^	10.98 ± 0.02^d^	20.1 ± 0.5^b^	46.2 ± 0.4^c^	19.04 ± 0.22^c,d^	246 ± 1^a^
CM_111_	2.8 ± 0.1^d^	12.6 ± 0.2^a^	20.9 ± 0.1^a^	45.6 ± 0.3^d^	18.1 ± 0.3^e^	239 ± 1^c^
CM_211_	2.5 ± 0.04^f^	11.5 ± 0.2^c^	20.9 ± 0.2^a^	46 ± 1^c^	19 ± 1^d,e^	236 ± 1^d^
CM_222_	3.1 ± 0.1^b^	10.6 ± 0.1^e^	21 ± 0.3^a^	47 ± 1^a^	18 ± 1^e^	237 ± 2^d^
CM_311_	2.21 ± 0.04^g^	11.1 ± 0.3^d^	19.5 ± 0.5^c,d^	46.88 ± 0.02^b^	20.3 ± 0.2^b^	239 ± 2^c^
CM_322_	2.6 ± 0.1^e^	9.8 ± 1.2^f^	19 ± 1^d^	45.5 ± 0.1^d^	23.04 ± 0.39^a^	246 ± 2^a^
CM_333_	2.9 ± 0.1^c^	11.8 ± 0.1^b^	20 ± 1^c^	46.2 ± 0.4^c^	20 ± 1^c^	244 ± 4^b^
*2nd Harvest*						
CM_0_	2.9 ± 0.1^d^	14.6 ± 0.3^d^	19.8 ± 0.1^a^	48 ± 1^b^	14 ± 1^c^	239 ± 2^e^
CM_111_	2.8 ± 0.1^e^	14.26 ± 0.02^e^	19.2 ± 0.2^b^	46 ± 1^d^	17.3 ± 0.4^a^	244.3 ± 0.2^d^
CM_211_	2.7 ± 0.1^e^	14.9 ± 0.3^c^	19.6 ± 0.4^a^	46 ± 0.5^d^	16.9 ± 0.4^a^	244 ± 2^d^
CM_222_	2.88 ± 0.03^d^	15.4 ± 0.1^b^	18 ± 1^c^	48 ± 1^b,c^	15.6 ± 0.2^b^	246.1 ± 1^c^
CM_311_	3.5 ± 0.1^b^	15.4 ± 0.2^b^	18.1 ± 0.4^c^	47.5 ± 0.1^c^	15.5 ± 0.3^b^	250 ± 1^b^
CM_322_	4.32 ± 0.12^a^	15.47 ± 0.2^b^	18.27 ± 0.04^c^	47.7 ± 0.1^b,c^	14.2 ± 0.2^c^	253.1 ± 0.4^a^
CM_333_	3.1 ± 0.1^c^	16.21 ± 0.03^a^	19.2 ± 0.3^b^	49 ± 1^a^	12 ± 2^d^	240 ± 3^d^
*3^rd^ Harvest*						
CM_0_	3.13 ± 0.1^f^	11.7 ± 0.2^b^	20.34 ± 0.01^a^	47.64 ± 0.04^c^	17.2 ± 0.2^d^	239 ± 1^g^
CM_111_	3.2 ± 0.1^f^	10.6 ± 0.2^c^	20 ± 1^b^	47.5 ± 0.1^c^	19.1 ± 1^c^	242 ± 2^f^
CM_211_	3.903 ± 0.044^d^	11.9 ± 0.3^b^	17.79 ± 0.01^d^	46.3 ± 0.3^d^	20 ± 1ª^,b^	256 ± 1^d^
CM_222_	3.3 ± 0.1^e^	11.8 ± 0.3^b^	19.3 ± 0.1^c^	45.3 ± 0.2^e^	20.3 ± 0.4^a^	248.9 ± 0.4^e^
CM_311_	4.1 ± 0.1^c^	13.3 ± 0.4^a^	16.4 ± 0.3^e^	47 ± 1^d^	19.5 ± 0.5^b,c^	261 ± 3^c^
CM_322_	6.02 ± 0.07^b^	13.2 ± 0.3^a^	16.5 ± 0.2^e^	50.1 ± 0.2^a^	14 ± 1^f^	264 ± 1^b^
CM_333_	7.6 ± 0.1^a^	13.22 ± 0.01^a^	15.5 ± 0.4^f^	48 ± 1^b^	15 ± 1^e^	280 ± 3^a^
Mean values (range of variability) of the centesimal composition						
CM_0_	3.2 (3.13–3.7)	12 (10.98–14.6)	20.1 (19.8–20.34)	47 (46.2–48)	17 (14–19.04)	241 (239–241)
CM_111_	2.9 (2.8–3.2)	12 (10.6–14.26)	20 (19.2–20.9)	46 (45.6–47.5)	18 (17.3–19.1)	242 (239–244.3)
CM_211_	3 (2.5–3.9)	13 (11.5–14.9)	19 (17.79–20.9)	46.1 (46–46.3)	19 (16.3–20)	245 (236–256)
CM_222_	3.1 (2.88–3.3)	13 (10.6–15.4)	19 (18–21)	47 (45.3–48)	18 (15.6–20.3)	244 (237–248.9)
CM_311_	3 (2.21–4.1)	13 (11.1–15.4)	18 (16.4–19.5)	47.1 (46.88–47.5)	18 (15.5–20.3)	250 (239–261)
CM_322_	4 (2.6–6.02)	13 (9.8–15.47)	18 (16.5–19)	48 (45.5–50.1)	17 (14–23.04)	254 (246–264)
CM_333_	5 (2.9–7.6)	14 (11.8–16.21)	18 (15.5–20)	48 (46.2–49)	16 (12–20)	255 (240–280)

In each harvest, values within the same column that are labelled with different lowercase letters differ significantly (*p* < 0.05), as determined by Tukey’s honest significant difference (HSD) test.

A similar nutritional profile of *C. maritimum* leaves has also been reported by Renna (39), who suggested a similar content of protein, fat, and ash, whereas higher and lower amounts of carbohydrates and fibre, respectively, were detected compared to our study. The effect of nutrient inputs on the nutritional value of wild edible species has also been confirmed by Polyzos et al. (23) and Paschoalinotto et al. (24) in *C. spinosum* and *S. hispamicus* plants, respectively, grown as annuals for a single growing period. According to these authors, ash and protein content may increase when low inputs are applied, whereas the increase in carbohydrate and energy content is associated with the application of higher amounts of N, P, K in the nutrient solution. Similar findings were also recorded in our study, but only for the first harvest, whereas the fat, protein, fibre, and energy content were the highest in successive harvests when high inputs were implemented.

Martins-Noguerol et al. (1) suggested that nutritional value of *C. maritimum* leaves is affected by growing conditions and agronomic practices, as they noted significant differences in total proteins and lipids content among wild plants collected from various regions, plants grown under optimum conditions in the greenhouse, and field grown plants, suggesting that the highest content of proteins and lipids was recorded in plants grown in the greenhouse under optimum conditions. Moreover, Martins-Noguerol et al. (20) reported that leaf protein content in *C. maritimum* plants collected from the wild was the highest in regions were soil was less fertile, whereas lipid content was not strongly affected by soil fertility. This finding could be associated with the up-regulation of stress-related proteins that help plants to cope with low nutrient availability, or the induction of specific pathways, while genotypic differences and adaptation mechanisms could also be the reason for this response in *C. maritimum* plants (40). Regarding the carbohydrate content, Hulkko et al. (41) reported higher values for plants grown under varied salinity levels, probably due to differences in sample preparation (fresh juice of leaves and fibre residue vs. freeze-dried leaves in our study). Moreover, Sánchez-Faure et al. (42) also suggested a higher content of carbohydrate in a dry weight basis in *C. maritimum* plants collected from the wild (69.5% vs. 14.0–23.4% in our study) combined with a significantly lower content of ash (11.6% vs. 15.5–21.0% in our study), while fat, protein and fibre content were in a similar range. Although no particular details are available in the study of Sánchez-Faure et al. (42), these differences could probably be attributed to differences in harvesting stage and date, since late harvest is commonly associated with higher carbohydrate and lower ash content (43, 44), while harvesting date and environmental conditions may also affect their content in a species dependent manner (1, 20, 45).

### 3.3 Hydrophilic compounds composition

The content of hydrophilic components (free sugars and organic acids) is presented in Table 3. A variable profile of free sugars was detected depending on the harvest and fertilization regime. Glucose was the major compound in all harvests, followed by fructose and trehalose in the 1st harvest, while sucrose, which was not detected in the 1st harvest, was found in significant amounts in the following harvests, followed by fructose and trehalose (detected only in the 3rd harvest). Moreover, the content of all detected sugars was higher for high nutrient inputs (CM_322_ and CM_333_, 300:200:200 mg/mL N:P:K and 300:300:300 mg/mL N:P:K, respectively) in the 2nd and 3rd harvests, whereas the control treatment and low inputs (CM_111_, 100:100:100 mg/mL N:P:K) showed the lowest content of individual and total sugars in the same harvests. Finally, the content of glucose fluctuated across the fertilization treatments in the first two harvests and showed its highest content in the 3rd harvest, regardless of the nutrient inputs. A similar trend was recorded for trehalose content, which increased in the 3rd harvest compared to the 1st one, especially when high nutrient inputs were administered. In contrast, the content of fructose and sucrose increased from the 1st to 2nd harvest, followed by a decrease in the 3rd harvest.

**Table 3:** Hydrophilic constituents (saccharides and organic acids) of *Crithmum maritimum* aerial parts (mean ± SD; *n* = 3).

	Saccharides (g 100/g dw)					Low molecular weight organic acids (mg 100/g dw)				
	Fructose	Glucose	Sucrose	Trehalose	Total	Oxalic	Quinic	Malic	Succinic	Total
*1st Harvest*										
CM_0_	1.2 ± 0.1^a^	3.5 ± 0.1^b^	n.d	0.74 ± 0.04^a^	5.5 ± 0.2^a^	5.504 ± 0.012^e^	6.6 ± 0.1^d^	3.5 ± 0.1^e^	3.6 ± 0.1^a^	19.1 ± 0.3^e^
CM_111_	0.93 ± 0.02^d^	2.9 ± 0.1^e^	n.d	0.41 ± 0.01^d^	4.3 ± 0.1^d^	6.36 ± 0.01^b^	7.7 ± 0.1^a^	4.6 ± 0.1^d^	3.6 ± 0.1^a^	22.2 ± 0.3^a^
CM_211_	0.83 ± 0.02^f^	2.6 ± 0.1^f^	n.d	0.31 ± 0.01^e^	3.7 ± 0.1^e^	5.52 ± 0.01^d^	7.3 ± 0.1^c^	4.7 ± 0.1^c^	1.86 ± 0.01^c^	19.4 ± 0.2^d^
CM_222_	1.02 ± 0.01^c^	3.7 ± 0.1^a^	n.d	0.41 ± 0.01^d^	5.1 ± 0.1^b^	6.44 ± 0.01^a^	5.1 ± 0.1^e^	2.6 ± 0.1^f^	1.64 ± 0.04^d^	15.7 ± 0.2^f^
CM_311_	1.023 ± 0.003^c^	3.2 ± 0.2^c^	n.d	0.69 ± 0.03^b^	4.9 ± 0.2^c^	5.73 ± 0.01^c^	7.4 ± 0.1^c^	4.6 ± 0.1^d^	1.89 ± 0.04^b,c^	19.6 ± 0.2^c^
CM_322_	1.047 ± 0.004^b^	3.5 ± 0.2^b^	n.d	0.41 ± 0.02^d^	4.9 ± 0.2^c^	4.66 ± 0.01^g^	7.4 ± 0.1^c^	5.7 ± 0.1^a^	1.99 ± 0.05^b^	19.8 ± 0.2^b^
CM_333_	0.89 ± 0.01^e^	2.93 ± 0.03^d^	n.d	0.496 ± 0.02^c^	4.31 ± 0.02^d^	4.93 ± 0.005^f^	7.49 ± 0.05^b^	5.27 ± 0.04^b^	1.91 ± 0.05^b^	19.6 ± 0.1^c^
*2nd Harvest*										
CM_0_	1.03 ± 0.02^e^	2.3 ± 0.1^e^	2.3 ± 0.1^c^	n.d	5.6 ± 0.2^e^	4.55 ± 0.02^f^	4.16 ± 0.04^f^	1.8 ± 0.1^e^	2.8 ± 0.1^a^	13.4 ± 0.2^f^
CM_111_	0.968 ± 0.002^f^	1.802 ± 0.008^f^	1.77 ± 0.04^f^	n.d	4.54 ± 0.04^f^	4.48 ± 0.01^g^	5.1 ± 0.1^e^	3.25 ± 0.03^d^	2.1 ± 0.1^d^	14.9 ± 0.2^e^
CM_211_	1.48 ± 0.04^b^	2.75 ± 0.01^c^	2.1 ± 0.1^e^	n.d	6.4 ± 0.1^c^	6.59 ± 0.02^c^	5.6 ± 0.2^d^	3.7 ± 0.1^c^	2.3 ± 0.1^b^	18.3 ± 0.5^d^
CM_222_	1.48 ± 0.01^b^	2.9 ± 0.1^b^	2.325 ± 0.004^c^	n.d	6.7 ± 0.1^b^	4.61 ± 0.005^e^	7.6 ± 0.1^a^	4.4 ± 0.1^b^	2.15 ± 0.02^c^	18.8 ± 0.3^c^
CM_311_	1.22 ± 0.01^d^	2.76 ± 0.03^c^	2.19 ± 0.02^d^	n.d	6.17 ± 0.02^d^	7.88 ± 0.005^b^	6.1 ± 0.2^c^	3.7 ± 0.1^c^	1.95 ± 0.06^e^	19.6 ± 0.4^b^
CM_322_	1.289 ± 0.004^c^	2.5 ± 0.1^d^	2.4 ± 0.1^b^	n.d	6.24 ± 0.04^d^	4.65 ± 0.01^d^	7.3 ± 0.2^b^	5.7 ± 0.2^a^	1.97 ± 0.03^e^	19.6 ± 0.4^b^
CM_333_	1.6 ± 0.1^a^	3.1 ± 0.1^a^	2.7 ± 0.1^a^	n.d	7.4 ± 0.2^a^	8.44 ± 0.004^a^	5.7 ± 0.2^d^	3.7 ± 0.1^c^	2.3 ± 0.1^b^	20.1 ± 0.4^a^
*3rd Harvest*										
CM_0_	0.76 ± 0.001^f^	2.9 ± 0.2^e^	1.14 ± 0.04^e^	0.5 ± 0.004^f^	5.3 ± 0.2^f^	4.34 ± 0.01^f^	5.7 ± 0.1^g^	2.99 ± 0.13^e^	1.3 ± 0.1^e^	14.3 ± 0.2^f^
CM_111_	0.89 ± 0.005^d^	3.3 ± 0.2^d^	1.25 ± 0.03^d^	0.54 ± 0.02^d^	5.954 ± 0.202^e^	4.402 ± 0.042^e^	6.969 ± 0.296^f^	4.2 ± 0.2^d^	1.2 ± 0.1^f^	16.71 ± 0.01^e^
CM_211_	0.93 ± 0.02^c^	3.77 ± 0.01^c^	1.21 ± 0.04^d^	0.59 ± 0.02^c^	6.49 ± 0.05^c^	7.83 ± 0.04^b^	7.5 ± 0.1^d^	5.08 ± 0.03^b^	1.559 ± 0.004^d^	21.9 ± 0.1^c^
CM_222_	0.72 ± 0.02^g^	3.9 ± 0.1^c^	1.14 ± 0.04^e^	0.52 ± 0.03^e^	6.3 ± 0.1^d^	7.723 ± 0.002^c^	8.4 ± 0.1^b^	5.084 ± 0.003^b^	1.7 ± 0.1^c^	22.86 ± 0.03^b^
CM_311_	0.93 ± 0.02^b^	5.2 ± 0.2^b^	1.57 ± 0.01^b^	0.68 ± 0.02^a^	8.4 ± 0.2^b^	7.02 ± 0.01^d^	7.7 ± 0.2^c^	4.5 ± 0.2^c^	5.4 ± 0.1^a^	24.5 ± 0.3^a^
CM_322_	0.9 ± 0.1^e^	5.4 ± 0.2^b^	1.8 ± 0.2^a^	0.673 ± 0.003^a^	8.7 ± 0.4^a^	8.44 ± 0.01^a^	8.61 ± 0.04^a^	6.4 ± 0.3^a^	1.301 ± 0.017^e^	24.6 ± 0.4^a^
CM_333_	1.04 ± 0.06^a^	5.7 ± 0.4^a^	1.4 ± 0.1^c^	0.6415 ± 0.00495^b^	8.8 ± 0.5^a^	3.97 ± 0.01^g^	7.3 ± 0.1^e^	4.961 ± 0.004^b^	3.1 ± 0.1^b^	19.3 ± 0.2^d^
Mean values (range of variability) of the saccharides and low molecular weight organic acids										
CM_0_	1.00 (0.76–1.20)	2.90 (2.30–3.50)	1.147 (0–2.30)	0.413 (0–0.74)	5.46 (5.3–5.6)	4.80 (4.34–5.50)	5.49 (4.16–6.60)	2.76 (1.80–3.50)	2.57 (1.30–3.60)	15.60 (13.40–19.10)
CM_111_	0.93 (0.89–0.97)	2.67 (1.80–3.30)	1.007 (0–1.77)	0.317 (0–0.54)	4.93 (4.3–5.95)	5.08 (4.40–6.36)	6.59 (5.10–7.70)	4.02 (3.25–6.97)	2.30 (1.20–3.60)	17.94 (14.90–22.20)
CM_211_	1.08 (0.83–1.48)	3.04 (2.60–3.77)	1.103 (0–2.10)	0.300 (0–0.59)	5.56 (3.7–6.49)	6.65 (5.52–7.83)	6.79 (5.60–7.50)	4.49 (3.70–5.08)	1.92 (1.56–2.30)	19.87 (18.30–21.90)
CM_222_	1.07 (0.72–1.48)	3.50 (2.90–3.90)	1.155 (0–2.325)	0.310 (0–0.52)	6.03 (5.1–6.7)	7.60 (6.44–7.72)	6.97 (5.10–8.40)	4.03 (2.60–5.08)	1.83 (1.64–2.15)	19.12 (15.70–22.86)
CM_311_	1.06 (0.93–1.22)	3.72 (2.76–5.20)	1.253 (0–2.19)	0.457 (0–0.69)	6.49 (4.9–8.4)	6.21 (5.73–7.02)	7.07 (6.10–7.70)	4.27 (3.70–4.50)	2.88 (1.95–5.40)	21.23 (19.60–24.50)
CM_322_	1.08 (0.90–1.29)	3.80 (2.50–5.40)	1.400 (0–2.40)	0.361 (0–0.673)	6.61 (4.9–8.7)	7.18 (4.65–8.44)	7.77 (7.30–8.61)	5.17 (5.70–6.40)	1.75 (1.30–1.99)	21.33 (19.60–24.60)
CM_333_	1.17 (0.89–1.60)	3.91 (2.93–5.70)	1.367 (0–2.70)	0.379 (0–0.642)	6.84 (4.31–8.80)	5.78 (3.97–8.44)	6.83 (5.70–7.30	4.64 (3.70–4.96)	2.11 (1.90–3.10)	19.67 (14.30–20.10)

n.d., not detected. In each harvest, values within the same column that are labelled with different lowercase letters differ significantly (*p* < 0.05), as determined by Tukey’s honest significant difference (HSD) test.

A similar free sugar profile was suggested by Meot-Duros and Magné (46), who also found high amounts of sucrose and glucose in *C. maritimum* aerial parts collected from two natural habitats. Similarly, Sánchez-Faure et al. (42) reported that glucose and fructose were the major free sugars in the tender stems of wild *C. maritimum* plants, followed by three polyols (mannitol, myoinositol, and glycerol), whereas sucrose content was below the limit of detection. According to Ventura et al. (47), sugar content and composition play a significant role in *C. maritimum*, as sugars are part of the osmoprotective mechanisms that allow plants to grow under saline environments. However, according to the same authors, there is also significant variation among the different genotypes/ecotypes associated with their overall salt tolerance (47). Regarding the effect of the fertilization regime, Paschoalinotto et al. (24) reported that the content of the major sugars in the leaves of *S. hispanicus* remained at high levels when low or no nutrients were applied, while a contrasting trend was suggested by Polyzos et al. (23) for leaf sugar content of *C. spinosum*, where high nutrient inputs (300:200:200 mg/mL of N-P-K) resulted in the highest content for most of the individual compounds and total sugars. The study by Petropoulos et al. (48) also highlighted the effect of N supply and harvesting time on free sugar composition in *Centaurea raphanina* subsp. *maxima* leaves, while the growing season and N form may also regulate sugar composition and their overall content (21). According to the literature, the content of free sugars may increase during the first growth stages of plants under N deprivation (49), especially in the nitrate form, while later on free sugars act as signalling molecules that regulate the expression of genes involved in photosynthesis process and plant metabolism as a response to plant’s nutrient status (50). Moreover, the harvesting stage and the balance between source (photosynthetic leaves) and sink (growing tissues) activities may regulate leaf sugars composition through their transport from mature to growing leaves (51). Therefore, it could be suggested that the continuous supplementation of *C. maritimum* plants with high nutrient inputs in our study is associated with increased sink activity and sugar accumulation in the growing tissues due to high nutrient availability (49).

Regarding the organic acids profile, quinic and oxalic acid were the major compounds in all samples, while lesser amounts of succinic and malic acid were also detected (Table 3). The effects of the fertilization regime across harvests were inconsistent. In particular, quinic and succinic acid levels were increased by low input fertilization (CM_0_ and CM_111_, no fertilizers added and 100:100:100 mg/mL N:P:K, respectively) in the 1st harvest, while higher input fertilization (CM_311_ and CM_322_, 300:100:100 mg/mL N:P:K and 300:200:200 mg/mL N:P:K, respectively) increased their levels through the 3rd harvest. In contrast, oxalic and malic acid levels were higher for moderate to high nutrient inputs (CM_311_ and CM_322_, 300:100:100 mg/mL N:P:K and 300:200:200 mg/mL N:P:K, respectively) across the harvests. Meot-Duros and Magné (46) reported the presence of maleic and quinic acid in methanolic extracts of *C. maritimum* aerial parts but did not detect the other compounds identified in our study. Quinic acid was also suggested by Gnocchi et al. (52) in ethyl acetate leaf extracts. Despite the lack of information regarding the organic acid composition of the species, the contradictions among literature reports could be partly attributed to the implementation of different extraction protocols (53) and availability of standards in chromatography methods. Moreover, the total amounts of available macronutrients and their respective ratios as applied through the studied nutrient solutions may result in synergism or antagonism among the nutrients, affecting their uptake and assimilation and consequently affecting plant physiology and metabolism (54). According to Petropoulos et al. (21), the nitrogen source has multiple impacts on organic acid composition, since nitrate is involved in organic acid production through the reduction of nitrate by nitrate reductase, in reduction in oxalic acid biosynthesis through the inhibition of the activity of oxalic acid oxidase, and the induction of organic acid synthesis as precursors of amino acids production. Moreover, N-P-K fertilization and the ratio of macronutrients may affect organic acid composition, particularly malic acid content, due to improved photosynthetic activity, increased tricarboxylic acid (TCA) cycle activation, and enhanced vacuolar malate transport and storage (55).

### 3.4 Lipophilic compounds composition

Table 4 presents the content of lipophilic compounds (tocopherols and main fatty acids) affected by the fertilization regime. The only tocopherol detected in all samples was *α*-tocopherol, with the highest content when plants were treated with CM_322_, regardless of the harvest. In contrast, low (CM_0_-CM_211_, control treatment and up to 200:100:100 mg/mL N:P:K) or very high (CM_333_, 300:300:300 mg/mL N:P:K) inputs resulted in low α-tocopherol content in the 2nd and 3rd harvests. Similar to our study, Nartea et al. (56) suggested that *α*-tocopherol was the main isoform of vitamin E identified in acetonic extracts of leaves of wild ecotypes of *C. maritimum,* with amounts varying between 52.8 mg/100 g dw and 1.9 mg/100 g dw. Moreover, the same authors detected lower amounts of *γ*-tocopherol among the various ecotypes (1.5 mg/100 g dw to 3.2 mg/100 g dw). Similarly, Generalić Mekinić et al. (57) reported that α-tocopherol was the main compound in *C. maritimum* leaf samples collected from 10 locations along the coastline and islands of Croatia, followed by *β*- and γ-tocopherol. The contrasting results in the profile of tocopherols could be explained by the different extraction protocols and analytical equipment used in previous studies (58, 59). Moreover, nutrient solution composition may affect the composition and profile of tocopherols, as reported by Polyzos et al. (23) and Paschoalinotto et al. (24) for *C. spinosum* and *S. hispanicus* plants, respectively. Interestingly, in both studies, the highest amounts of *α*- and total tocopherols were detected when *C. spinosum* and *S. hispanicus* plants were fertigated with a nutrient solution containing 300:200:200 mg/mL of N:P:K, which is fully aligned with our study. Therefore, it can be suggested that high amounts of N and a balanced content of P and K are most beneficial for tocopherol biosynthesis. However, in addition to nutrient inputs, other factors such as the nitrogen source, harvesting time, and successive harvesting may affect tocopherol biosynthesis and accumulation (38).

**Table 4:** Lipophilic constituents (tocopherols and fatty acids) of *Crithmum maritimum* aerial parts (mean ± SD; *n* = 3).

	Tocopherol isoform (mg 100/g dw)	High molecular weight organic acids (%)										
	*α*-Tocopherol	C12:0	C16:0	C16:1	C18:0	C18:1n9c	C18:2n6c	C18:3n3	C20:0	SFA	MUFA	PUFA
*1st Harvest*												
CM_0_	0.1 ± 0.01^e^	1.445 ± 0.001^a^	24.87 ± 0.01^d^	1.995 ± 0.001^g^	3.64 ± 0.02^b^	4.84 ± 0.01^d^	28.697 ± 0.002^c^	25.03 ± 0.01^c^	1.88 ± 0.01^a^	39 ± 0.1^a,b^	1.99 ± 0.02^d^	59.02 ± 0.03^b,c^
CM_111_	0.081 ± 0.003^f^	0.93 ± 0.03^f^	25.9 ± 0.7^b^	2.34 ± 0.02^b^	3.313 ± 0.003^e^	4.8 ± 0.1^d^	28 ± 0.3^d^	26.8 ± 0.7^a^	1.53 ± 0.04^d^	38 ± 1^d^	2.3 ± 0.7^b^	59.98 ± 0.33^a^
CM_211_	0.0682 ± 0.0003^g^	1.16 ± 0.01^d^	27.01 ± 0.12^a^	2.69 ± 0.02^a^	3.24 ± 0.06^f^	4.83 ± 0.04^d^	27.7 ± 0.1^e^	25.81 ± 0.04^b^	1.25 ± 0.02^f^	38.6 ± 0.3^b,c^	2.69 ± 0.02^a^	58.7 ± 0.2^c^
CM_222_	0.1293 ± 0.0003^d^	1.09 ± 0.02^e^	25.76 ± 0.04^b,c^	2.15 ± 0.03^e^	3.76 ± 0.01^a^	5.01 ± 0.03^c^	29.04 ± 0.09^b^	24.2 ± 0.04^d^	1.815 ± 0.004^b^	39.3 ± 0.2^a^	2.2 ± 0.1^c^	58.6 ± 0.1^c^
CM_311_	0.21 ± 0.01^b^	1.16 ± 0.04^d^	25.1 ± 0.8^d^	2.09 ± 0.04^f^	3.6 ± 0.2^b^	5.6 ± 0.2^a^	27.48 ± 0.2^f^	26 ± 1^b^	1.74 ± 0.06^c^	39 ± 1^b,c^	2 ± 1^d^	59.2 ± 0.6^b^
CM_322_	0.241 ± 0.004^a^	1.29 ± 0.02^c^	25.48 ± 0.23^c^	2.21 ± 0.04^d^	3.51 ± 0.04^c^	5.45 ± 0.11^b^	30.4 ± 0.4^a^	23.1 ± 0.4^e^	1.51 ± 0.08^d^	39 ± 01^b,c^	2.2 ± 0.2^c^	59.2 ± 0.6^b^
CM_333_	0.173 ± 0.002^c^	1.32 ± 0.06^b^	25.7 ± 0.7^b,c^	2.25 ± 0.02^c^	3.38 ± 0.04^d^	4.68 ± 0.02^e^	29.1 ± 0.2^b^	25.3 ± 0.7^c^	1.31 ± 0.01^e^	38 ± 1^c^	2.2 ± 1^c^	59.5 ± 0.3^b^
*2nd Harvest*												
CM_0_	0.037 ± 0.001^f^	0.45 ± 0.002^a^	20.88 ± 0.03^b^	2.98 ± 0.01^e^	3.9 ± 0.1^a^	2.132 ± 0.004^b^	25.27 ± 0.02^f^	33.65 ± 0.01^g^	2.32 ± 0.04^a^	35.5 ± 0.2^a^	2.977 ± 0.04^e^	61.6 ± 0.1^e^
CM_111_	0.057 ± 0.001^c^	0.33 ± 0.01^c^	20.5 ± 0.1^d^	2.9 ± 0.1^f^	3.42 ± 0.03^c^	1.89 ± 0.01^c^	25.7 ± 0.5^e^	35.7 ± 0.3^b^	2.1 ± 0.01^b^	34 ± 1^c^	2.9 ± 0.1^f^	63.8 ± 0.5^c^
CM_211_	0.039 ± 0.001^e,f^	0.31 ± 0.02^d^	20.78 ± 0.1^c^	3.22 ± 0.04^b^	2.97 ± 0.05^e^	1.8 ± 0.1^d^	29.09 ± 0.01^a^	34.4 ± 0.1^e^	1.7 ± 0.1^c^	31.5 ± 0.4^e^	3.22 ± 0.04^b^	65.7 ± 0.2^a^
CM_222_	0.0502 ± 0.0014^d^	0.36 ± 0.02^b^	22.37 ± 0.04^a^	3.15 ± 0.01^c^	3.49 ± 0.01^b^	2.14 ± 0.01^b^	25.9 ± 0.2^d^	33.8 ± 0.1^f^	1.7 ± 0.1^c^	34.7 ± 0.3^b^	3.2 ± 0.1^c^	62.3 ± 0.2^d^
CM_311_	0.1498 ± 0.0024^b^	0.358 ± 0.004^b^	20.3 ± 0.1^e^	3.08 ± 0.03^d^	3.2 ± 0.1^d^	1.72 ± 0.04^e^	26.6 ± 0.1^c^	37.1 ± 0.1^a^	1.7 ± 0.1^c^	31.5 ± 0.3^e^	3.1 ± 0.1^d^	65.8 ± 0.2^a^
CM_322_	0.25 ± 0.01^a^	0.45 ± 0.01^a^	20.9 ± 0.1^b^	3.52 ± 0.03^a^	3.1 ± 0.1^d^	1.88 ± 0.1^c^	27.72 ± 0.04^b^	34.5 ± 0.1^d^	1.37 ± 0.04^e^	32.3 ± 0.3^d^	3.5 ± 0.1^a^	64.5 ± 0.2^b^
CM_333_	0.041 ± 0.001^e^	0.35 ± 0.01^b^	20.52 ± 0.03^d^	2.9 ± 0.1^f^	3.14 ± 0.01^d^	2.19 ± 0.01^a^	27.73 ± 0.04^b^	35.22 ± 0.02^c^	1.49 ± 0.02^d^	31.6 ± 0.3^e^	2.86 ± 0.05^f^	65.6 ± 0.1^a^
*3rd Harvest*												
CM_0_	1.737 ± 0.003^b^	n.d	40.3 ± 0.7^a^	n.d	8.4 ± 0.2^a^	6.1 ± 0.1^a^	24.4 ± 0.5^f^	20.8 ± 0.1^f^	n.d	48.7 ± 0.5^a^	6.1 ± 0.1^d^	45.2 ± 0.5^g^
CM_111_	0.13 ± 0.01^g^	3.004 ± 0.049^c^	32.7 ± 0.2^b^	3.38 ± 0.04^a^	6.2 ± 0.1^b^	4.02 ± 0.05^c^	25.02 ± 0.31^e^	22.2 ± 0.3^e^	3.42 ± 0.05^b^	45.3 ± 0.1^b^	7.4 ± 0.1^a^	47.27 ± 0.03^f^
CM_211_	0.17 ± 0.01^f^	3.18 ± 0.02^b^	30.3 ± 0.1^c^	3.01 ± 0.09^b^	5.9 ± 0.1^c^	4.3 ± 0.1^b^	26.5 ± 0.3^d^	24.6 ± 0.3^b^	2.14 ± 0.05^e^	41.6 ± 0.1^e^	7.297 ± 0.004^b^	51.1 ± 0.1^d^
CM_222_	0.39 ± 0.01^d^	2.4 ± 0.1^d^	33 ± 1^b^	2.9 ± 0.1^c^	6.4 ± 0.2^b^	3.2 ± 0.1^f^	25.17 ± 0.04^e^	23.62 ± 0.04^d^	3.6 ± 0.1^a^	45.1 ± 0.3^c^	6.1 ± 0.2^d^	48.8 ± 0.1^e^
CM_311_	0.902 ± 0.004^c^	2.2 ± 0.1^e^	29.4 ± 0.2^d^	2.5 ± 0.1^e^	5.5 ± 0.1^d^	3.4 ± 0.1^e^	26.9 ± 0.4^c^	27.7 ± 0.2^a^	2.3 ± 0.1^d^	39.5 ± 0.2^f^	5.94 ± 0.01^e^	54.6 ± 0.2^b^
CM_322_	1.96 ± 0.01^a^	3.3 ± 0.1^a^	30.2 ± 0.1^c^	2.7 ± 0.1^d^	5.3 ± 0.3^e^	3.4 ± 0.2^e^	28 ± 0.3^b^	24.1 ± 0.2^c^	2.9 ± 0.1^c^	41.7 ± 0.1^d^	6.09 ± 0.04^d^	52.18 ± 0.03^c^
CM_333_	0.203 ± 0.003^e^	1.93 ± 0.01^f^	28.02 ± 0.01^e^	2.88 ± 0.04^c^	4.9 ± 0.1^f^	3.7 ± 0.1^d^	28.7 ± 0.1^a^	27.8 ± 0.1^a^	2.13 ± 0.02^e^	36.9 ± 0.1^g^	6.55 ± 0.04^c^	56.5 ± 0.03^a^
Mean values (range of variability) of the tocopherol isoforms and high molecular weight organic acids												
CM_0_	0.625 (0.037–1.737)	1.03 (0.45–1.45)	28.7 (20.9–40.3)	1,66 (0–2.98)	5.31 (3.64–8.40)	4.36 (2.13–6.10)	26.8 (24.4–28.7)	26.08 (20.8–33.65)	1.75 (1.25–2.32)	41.10 (35.5–48.7)	3.36 (1.99–6.10)	54.94 (45.2–61.6)
CM_111_	0.089 (0.057–0.130)	1.42 (0.33–3.00)	26.9 (20.5–32.7)	2.87 (2.34–3.38)	4.31 (3.31–6.20)	3.57 (1.89–4.02)	26.2 (25.0–28.0)	27.20 (22.2–35.7)	1.78 (1.53–2.10)	40.90 (34.0–45.3)	4.20 (2.30–7.40)	57.02 (47.3–63.8)
CM_211_	0.092 (0.039–0.170)	1.55 (0.31–3.18)	26.0 (20.8–30.3)	2.97 (2.69–3.01)	4.04 (2.97–5.90)	3.64 (1.80–4.30)	27.8 (25.0–29.1)	28.27 (24.6–34.4)	1.55 (1.25–1.70)	37.20 (31.5–41.6)	4.40 (2.69–7.30)	58.50 (51.1–65.7)
CM_222_	0.190 (0.0502–0.390)	1.28 (0.36–2.40)	27.0 (22.4–33.0)	2.98 (2.15–2.90)	4.55 (3.49–6.40)	3.78 (2.14–3.20)	26.7 (25.2–29.0)	27.20 (23.6–33.8)	2.37 (1.70–3.60)	39.70 (34.7–45.1)	3.83 (2.20–6.10)	56.56 (48.8–62.3)
CM_311_	0.421 (0.1498–0.902)	1.24 (0.358–2.20)	25.6 (20.3–29.4)	2.56 (2.09–2.50)	4.10 (3.20–5.50)	3.57 (1.72–3.40)	26.9 (26.6–27.5)	30.60 (27.7–37.1)	1.91 (1.70–2.30)	36.70 (31.5–39.5)	4.33 (2.00–5.94)	59.20 (54.6–65.8)
CM_322_	0.817 (0.241–1.960)	1.68 (0.45–3.30)	25.5 (20.9–30.2)	2.80 (2.21–2.70)	3.97 (3.10–5.30)	3.57 (1.88–3.40)	28.7 (27.7–30.4)	27.23 (24.1–34.5)	1.59 (1.37–1.80)	37.70 (32.3–41.7)	3.93 (2.20–6.09)	58.63 (52.2–64.5)
CM_333_	0.139 (0.041–0.203)	1.20 (0.35–1.93)	24.8 (20.5–28.0)	2.67 (2.25–2.88)	3.81 (3.14–4.90)	3.86 (2.19–3.70)	28.5 (27.7–29.1)	29.61 (25.2–35.2)	1.64 (1.31–2.13)	35.50 (31.6–36.9)	4.34 (2.20–6.55)	60.53 (56.5–65.6)

Some percentages below 2% are not shown. In each harvest, values within the same column that are labelled with different lowercase letters differ significantly (*p* < 0.05), as determined by Tukey’s honest significant difference (HSD) test.

The major fatty acids in all samples were palmitic, linoleic, and α-linolenic acids, which were detected in amounts that varied among the different fertilization regimes (Table 4). In particular, no significant variation in the content of the three major acids was observed at the 1st harvest, whereas α-linolenic and palmitic acid contents were significantly increased in the 2nd and 3rd harvests, respectively, regardless of the fertilization regime. This trend was also reflected in the overall content of saturated (SFA) and polyunsaturated fatty acids (PUFA), which were the major classes of fatty acid compounds. Moreover, a variable effect of nutrient solution composition on the fatty acid profile was observed across the harvests, although it seems that high nutrient inputs benefited the synthesis of the major unsaturated fatty acids and reduced the content of palmitic acid. In the first two harvests, PUFA was the major class of fatty acids, followed by monounsaturated (MUFA) and SFA, whereas PUFA and SFA were detected in similar amounts in the 3rd harvest, but only when low to moderate nutrient inputs were administered (CM_0_ to CM_222_, no fertilizers added and up to 200:200:200 mg/mL N:P:K). The values of PUFA/SFA and omega-6/omega-3 fatty acids were higher than 0.45 and lower than 4.0, respectively, for all samples. However, a varied response was recorded depending on the fertilization regime and harvest, with samples from the 2nd harvest having the most nutritious profile of fatty acids (PUFA values between 1.7 and 2.1 and omega-6/omega-3 values between 0.72 and 0.84).

A similar profile of fatty acids was reported by Ventura et al. (47) who also detected significant amounts of palmitic, linoleic, and α-linolenic acids in the aerial parts of four distinct genotypes of pot-grown *C. maritimum* plants, while the same compounds were identified by Maoloni et al. (60) and Guil-Guerrero and Rodríguez-García (61) in fresh sprouts and tender leaves of the plant, respectively. The same major compounds were suggested by Martins-Noguerol et al. (1) and Castillo et al. (5), who additionally detected high amounts of petroselinic acid. The fertilization regime may regulate the content and profile of fatty acids, especially the form and rate of nitrogen, which affect the content of fatty acids, such as palmitic, linoleic, and α-linolenic acid (19). Moreover, environmental conditions, especially abiotic stressors such as drought and salinity, can also affect the fatty acid profile as part of plant defense mechanisms, where SFA content usually increases at the expense of PUFA (62). This finding was also observed in our study, where plants treated with the control treatment showed increased SFA content in the 2nd and 3rd harvest, followed by a concomitant decrease in the content of linoleic and α-linolenic acid.

### 3.5 Phenolic compounds composition

The profile of phenolic compounds observed in the hydroethanolic extracts of the aerial parts of *Crithmum maritimum* in the present study is shown in the Supplementary Material (Supplementary Figure S1), while the criteria evaluated for the provisional identification of these constituents and their respective quantifications are presented in Tables 5, 6, respectively. Twelve phenolic compounds were tentatively identified in total, including five phenolic acids (peaks 1, 2, 4–6) and seven flavonoids (peaks 3, 7–12), detected in amounts that varied among the samples. The most abundant compounds were two phenolic acids, namely, *cis*-5-*O*-*p*-Coumaroylquinic acid and *trans*-*O*-Caffeoylquinic acid (peaks 4 and 2, respectively), in the 1st and 2nd harvests, respectively, regardless of the fertilization regime. In contrast, the major compounds detected in the 3rd harvest were flavonoids, such as Apigenin-6,8-dihexoside (peak 3) in the CM_211_ (200:100:100 mg/mL N:P:K) treatment and two isomeric forms of Luteolin-*O*-deoxyhexosylhexoside (peaks 9 and 10, respectively), for the other treatments. Moreover, the 2nd harvest showed the highest total phenol content regardless of the fertilization treatment, mostly because of the high amounts of *trans*-*O*-Caffeoylquinic acid, especially in the CM_322_ (300:200:200 mg/mL N:P:K) treatment, where the highest overall content of total phenol was recorded. For the 1st and 3rd harvests, treatment CM_111_ (100:100:100 mg/mL N:P:K) showed the highest content of total phenol and specific phenolic compounds, for example, peaks 1–6, 8, 9, 11, 12 in the 1st harvest and peaks 1, 6–8, 11, 12 in the 3^rd^ harvest.

**Table 5:** Criteria evaluated for the tentative identification of phenol constituents in the hydroethanolic extracts of *Crithmum maritimum* aerial parts.

Peak	R_t_	λ_max_	[M-H]^−^	MS^2^	Tentative identification
1	6.7	323	353	191 (100), 179 (35), 135 (5)	*cis-O*-caffeoylquinic acid
2	7.2	325	353	191 (100),179 (43), 135 (6)	*trans*-*O*-caffeoylquinic acid
3	9.82	333	593	503 (32), 473 (100), 353 (6)	Apigenin-6,8-dihexoside
4	11.84	312	337	191 (100), 163 (10)	*cis*-5-*O*-p-coumaroylquinic acid
5	13.13	310	337	191 (100), 163 (10)	*trans*-5-*O*-p-coumaroylquinic acid
6	13.69	325	367	191 (100), 193 (75)	5-*O*-feruloylquinic acid
7	17.49	354	609	301 (100)	Quercetin-*O*-deoxyhexosylhexoside
8	17.73	353	609	301 (100)	Quercetin-*O*-deoxyhexosylhexoside
9	18.08	342	593	285 (100)	Luteolin-*O*-deoxyhexosylhexoside
10	19.56	341	593	285 (100)	Luteolin-*O*-deoxyhexosylhexoside
11	22.07	339	623	315 (100)	Isorhamnetin-*O*-deoxyhexosylhexoside
12	24.05	346	607	299 (100), 283 (10)	Diosmetin-*O*-deoxyhexosylhexoside

Calibration curves: p-Coumaric acid (*y* = 301,950× + 6,966,7, *R*^2^ = 1, LOD = 0.68 μg/mL and LOQ = 1.61 μg/mL, peaks 4 to 5), Caffeic acid (*y* = 388,345× + 406,369, *R*^2^ = 0.9939, LOD = 8.57 μg/mL and LOQ = 25.97 μg/mL, peaks 1 to 2), Quercetin-3-O-glucoside (*y* = 34.843× – 160.173, *R*^2^ = 0.9998, LOD = 0.21 μg/mL; LOQ = 0.71 μg/mL, peaks 3, 7 to 12), Ferulic acid (*y* = 633,126× – 185,462, *R*^2^ = 0.999, LOD = 1.25 μg/mL; LOQ = 3.80 μg/mL, peak 6).

**Table 6:** Quantification of phenol constituents in hydroethanolic extracts of *Crithmum maritimum* aerial parts (mean ± SD; *n* = 3).

	Peaks												TPC
	1	2	3	4	5	6	7	8	9	10	11	12	
*1st Harvest*													
CM_0_	0.23 ± 0.01^b^	0.71 ± 0.01^b^	0.493 ± 0.003^d^	0.74 ± 0.01^e^	0.329 ± 0.002^e^	0.115 ± 0.001^c^	0.4593 ± 0.0002^d^	0.388 ± 0.001^d^	0.38015 ± 0.00002^d^	0.378 ± 0.001^d^	0.393 ± 0.001^d^	0.428 ± 0.003^d^	5.05 ± 0.01^c^
CM_111_	0.36 ± 0.01^a^	0.78 ± 0.03^a^	0.653 ± 0.001^a^	1.7594 ± 0.0004^a^	0.709 ± 0.01^a^	0.18 ± 0.01^a^	0.582 ± 0.001^b^	0.502 ± 0.001^a^	0.49 ± 0.001^a^	0.477 ± 0.001^b^	0.506 ± 0.001^a^	0.6037 ± 0.003^a^	7.61 ± 0.01^a^
CM_211_	0.17 ± 0.01^f^	0.261 ± 0.004^g^	0.4713 ± 0.0005^f^	0.86 ± 0.01^c^	0.359 ± 0.002^b^	0.091 ± 0.001^f^	0.3763 ± 0.0002^f^	0.3457 ± 0.0001^g^	0.3474 ± 0.0002^f^	0.3441 ± 0.0003^g^	0.3556 ± 0.0001^g^	0.39374 ± 0.00002^e^	4.375 ± 0.001^f^
CM_222_	0.203 ± 0.002^d^	0.38 ± 0.01^f^	0.477 ± 0.002^e^	0.804 ± 0.001^d^	0.342 ± 0.001^d^	0.103 ± 0.001^e^	0.404 ± 0.001^e^	0.353 ± 0.0001^f^	0.347 ± 0.0003^g^	0.35 ± 0.001^f^	0.369 ± 0.002^e^	0.377 ± 0.001^g^	4.51 ± 0.01^e^
CM_311_	0.21 ± 0.01^c^	0.463 ± 0.01^c^	0.599 ± 0.001^b^	0.91 ± 0.01^b^	0.35 ± 0.01^c^	0.117 ± 0.002^b^	0.597 ± 0.006^a^	0.495 ± 0.001^b^	0.487 ± 0.001^b^	0.4861 ± 0.0003^a^	0.5027 ± 0.0001^b^	0.5336 ± 0.001^c^	5.75 ± 0.01^b^
CM_322_	0.106 ± 0.002^g^	0.409 ± 0.001^e^	0.4194 ± 0.001^g^	0.52 ± 0.01^g^	0.203 ± 0.004^g^	0.081 ± 0.001^g^	0.372 ± 0.001^g^	0.3584 ± 0.0001^e^	0.3574 ± 0.0004^e^	0.3548 ± 0.0001^e^	0.35982 ± 0.000003^f^	0.3837 ± 0.0003^f^	3.923 ± 0.001^g^
CM_333_	0.19 ± 0.01^e^	0.42 ± 0.01^d^	0.545 ± 0.001^c^	0.63 ± 0.002^f^	0.2815 ± 0.0005^f^	0.107 ± 0.002^d^	0.4669 ± 0.0003^c^	0.4317 ± 0.0004^c^	0.43187 ± 0.00004^c^	0.4248 ± 0.0002^c^	0.4391 ± 0.0003^c^	0.546 ± 0.003^b^	4.912 ± 0.003^d^
*2nd Harvest*													
CM_0_	0.2996 ± 0.0096^c^	3.556 ± 0.001^c^	0.348 ± 0.0004^g^	0.554 ± 0.002^c^	0.32 ± 0.01^c^	0.191 ± 0.002^c^	0.569 ± 0.002^e^	0.304 ± 0.002^f^	0.276 ± 0.001^f^	0.293 ± 0.001^g^	0.283 ± 0.001^g^	0.4451 ± 0.0003^e^	7.43 ± 0.01^c^
CM_111_	0.75 ± 0.02^a^	6.9 ± 0.04^b^	0.625 ± 0.001^a^	1.35 ± 0.02^a^	0.713 ± 0.03^a^	0.391 ± 0.003^b^	0.87 ± 0.01^c^	0.65 ± 0.01^a^	0.551 ± 0.003^b^	0.53 ± 0.001^a^	0.4957 ± 0.0002^b^	0.8023 ± 0.0003^a^	14.626 ± 0.003^b^
CM_211_	0.184 ± 0.001^g^	1.507 ± 0.005^g^	0.349 ± 0.003^f^	0.39 ± 0.01^f^	0.191 ± 0.004^f^	0.121 ± 0.003^f^	0.485 ± 0.001^f^	0.304 ± 0.002^f^	0.29486 ± 0.00004^e^	0.299 ± 0.001^f^	0.2913 ± 0.0002^e^	0.3601 ± 0.001^f^	4.77 ± 0.01^g^
CM_222_	0.223 ± 0.001^e^	2.04 ± 0.01^e^	0.555 ± 0.001^c^	0.511 ± 0.004^d^	0.257 ± 0.008^d^	0.17211 ± 0.00003^d^	0.759 ± 0.01^d^	0.596 ± 0.001^c^	0.5196 ± 0.0003^c^	0.52 ± 0.001^b^	0.5033 ± 0.0001^a^	0.5664 ± 0.001^c^	7.23 ± 0.02^d^
CM_311_	0.282 ± 0.002^d^	2.12 ± 0.01^d^	0.3651 ± 0.0003^e^	0.52 ± 0.01^d^	0.254 ± 0.001^d^	0.15045 ± 0.00002^e^	0.44 ± 0.003^g^	0.349 ± 0.004^e^	0.2951 ± 0.0003^e^	0.301 ± 0.001^e^	0.287 ± 0.001^f^	0.325 ± 0.001^g^	5.68 ± 0.01^f^
CM_322_	0.465 ± 0.005^b^	8.73 ± 0.01^a^	0.572 ± 0.001^b^	1.04 ± 0.01^b^	0.56 ± 0.01^b^	0.425 ± 0.003^a^	0.92 ± 0.003^b^	0.641 ± 0.001^b^	0.48 ± 0.004^d^	0.461 ± 0.002^c^	0.454 ± 0.0004^c^	0.5364 ± 0.0001^d^	15.28 ± 0.03^a^
CM_333_	0.2074 ± 0.0005^f^	1.83 ± 0.01^f^	0.479 ± 0.002^d^	0.427 ± 0.002^e^	0.23 ± 0.01^e^	0.15 ± 0.01^e^	0.96 ± 0.01^a^	0.45 ± 0.003^d^	0.574 ± 0.003^a^	0.4346 ± 0.0004^d^	0.4343 ± 0.0003^d^	0.6175 ± 0.0004^b^	6.794 ± 0.001^e^
*3rd Harvest*													
CM_0_	0.345 ± 0.001^d^	0.064 ± 0.005^d^	0.549 ± 0.001^d^	0.128 ± 0.003^e^	0.26 ± 0.01^d^	0.16 ± 0.01^e^	0.4978 ± 0.0007^b^	0.4892 ± 0.0004^b^	0.55 ± 0.01^d^	0.516 ± 0.001^e^	0.509 ± 0.001^c^	0.506 ± 0.001^c^	4.58 ± 0.01^e^
CM_111_	0.401 ± 0.006^a^	0.083 ± 0.001^b^	0.5567 ± 0.0005^b^	0.216 ± 0.003^b^	0.42 ± 0.02^b^	0.207 ± 0.003^a^	0.4995 ± 0.0013^a^	0.504 ± 0.001^a^	0.569 ± 0.002^b^	0.648 ± 0.001^a^	0.5398 ± 0.0004^a^	0.5121 ± 0.001^b^	5.1577 ± 0.0002^a^
CM_211_	0.27 ± 0.01^e^	0.058 ± 0.004^f^	0.524 ± 0.001^f^	0.128 ± 0.004^e^	0.162 ± 0.001^f^	0.126 ± 0.002^f^	0.4836 ± 0.0004^d^	0.478 ± 0.001^e^	0.496 ± 0.001^f^	0.505 ± 0.001^g^	0.476 ± 0.001^f^	0.4711 ± 0.001^f^	4.176 ± 0.005^f^
CM_222_	0.37 ± 0.01^c^	0.066 ± 0.001^d^	0.5649 ± 0.0001^a^	0.1757 ± 0.0005^c^	0.23 ± 0.01^e^	0.169 ± 0.003^c^	0.484 ± 0.001^d^	0.48544 ± 0.00001^c^	0.601 ± 0.002^a^	0.64 ± 0.01^b^	0.535 ± 0.003^b^	0.51642 ± 0.00001^a^	4.84 ± 0.02^c^
CM_311_	0.267 ± 0.001^e^	0.072 ± 0.003^c^	0.544 ± 0.001^e^	0.149 ± 0.005^d^	0.343 ± 0.004^c^	0.162 ± 0.003^d^	0.4875 ± 0.0004^c^	0.4836 ± 0.0004^d^	0.5561 ± 0.0002^c^	0.556 ± 0.003^c^	0.497 ± 0.001^d^	0.49028 ± 0.0009^d^	4.608 ± 0.005^d^
CM_322_	0.2296 ± 0.0011^f^	0.062 ± 0.002^e^	0.518 ± 0.0003^g^	0.072 ± 0.001^f^	0.128 ± 0.004^g^	0.167 ± 0.002^c^	0.448 ± 0.001^f^	0.4416 ± 0.0002^f^	0.5234 ± 0.002^e^	0.534 ± 0.003^d^	0.446 ± 0.002^g^	0.41936 ± 0.0003^g^	3.99 ± 0.01^g^
CM_333_	0.384 ± 0.001^b^	0.101 ± 0.003^a^	0.555 ± 0.001^c^	0.449 ± 0.003^a^	0.438 ± 0.001^a^	0.192 ± 0.002^b^	0.4751 ± 0.0003^e^	0.4774 ± 0.0004^e^	0.571 ± 0.004^b^	0.5105 ± 0.0004^f^	0.4903 ± 0.0017^e^	0.4882 ± 0.0002^e^	5.131 ± 0.001^b^
Mean values (range of variability) of the phenol constituents													
CM_0_	0.29 (0.23–0.35)	1.44 (0.06–3.56)	0.46 (0.35–0.55)	0.47 (0.13–0.74)	0.30 (0.26–0.33)	0.16 (0.11–0.19)	0.51 (0.46–0.57)	0.39 (0.30–0.49)	0.40 (0.28–0.55)	0.39 (0.29–0.52)	0.39 (0.28–0.51)	0.46 (0.43–0.51)	5.69 (4.58–7.43)
CM_111_	0.50 (0.36–0.75)	2.59 (0.08–6.90)	0.61 (0.56–0.65)	0.78 (0.22–1.76)	0.51 (0.42–0.71)	0.26 (0.18–0.39)	0.65 (0.50–0.87)	0.55 (0.50–0.65)	0.54 (0.49–0.57)	0.55 (0.48–0.65)	0.51 (0.46–0.54)	0.64 (0.51–0.80)	9.13 (5.16–14.63)
CM_211_	0.21 (0.17–0.27)	0.61 (0.06–1.51)	0.45 (0.35–0.52)	0.46 (0.13–0.86)	0.24 (0.16–0.36)	0.11 (0.09–0.13)	0.45 (0.38–0.49)	0.38 (0.30–0.48)	0.38 (0.29–0.50)	0.38 (0.30–0.51)	0.37 (0.29–0.48)	0.41 (0.36–0.47)	4.44 (4.18–4.77)
CM_222_	0.27 (0.20–0.37)	0.83 (0.07–2.04)	0.53 (0.48–0.56)	0.50 (0.18–0.80)	0.28 (0.23–0.34)	0.15 (0.10–0.17)	0.55 (0.40–0.76)	0.48 (0.35–0.60)	0.49 (0.35–0.60)	0.50 (0.35–0.64)	0.47 (0.37–0.54)	0.49 (0.38–0.57)	5.53 (4.51–7.23)
CM_311_	0.25 (0.21–0.28)	0.88 (0.07–2.12)	0.50 (0.37–0.60)	0.53 (0.15–0.91)	0.32 (0.25–0.35)	0.14 (0.12–0.15)	0.51 (0.44–0.60)	0.44 (0.35–0.50)	0.45 (0.30–0.56)	0.45 (0.30–0.56)	0.43 (0.29–0.50)	0.45 (0.33–0.53)	5.35 (4.61–5.75)
CM_322_	0.27 (0.11–0.47)	3.07 (0.06–8.73)	0.50 (0.42–0.57)	0.54 (0.07–1.04)	0.30 (0.13–0.56)	0.22 (0.08–0.43)	0.58 (0.37–0.92)	0.48 (0.36–0.64)	0.45 (0.36–0.52)	0.45 (0.35–0.53)	0.42 (0.36–0.45)	0.45 (0.38–0.54)	7.73 (3.99–15.28)
CM_333_	0.26 (0.19–0.38)	0.78 (0.10–1.83)	0.53 (0.48–0.55)	0.50 (0.43–0.63)	0.32 (0.23–0.44)	0.15 (0.11–0.19)	0.63 (0.47–0.96)	0.45 (0.43–0.48)	0.53 (0.43–0.57)	0.46 (0.43–0.51)	0.48 (0.43–0.49)	0.55 (0.49–0.62)	5.61 (4.91–6.79)

TPC, total phenol constituents. In each harvest, values within the same column that are labelled with different lowercase letters differ significantly (*p* < 0.05), as determined by Tukey’s honest significant difference (HSD) test.

Similarly, Martins-Noguerol et al. (1) detected seven hydroxycinnamic acids in the hydromethanolic extracts of leaves of four wild ecotypes and one ecotype cultivated under field conditions, including caffeoylquinic, coumaroylquinic, and feruloylquinic acid, which were also found in our study, as well as four flavonoids, including quercetin derivatives that were also identified in the present work. Moreover, Martins-Noguerol et al. (1) suggested that flavonoids were the major class of polyphenols across the studied genotypes, mostly due to the high content of rutin, which was the most abundant polyphenol, whereas the cultivated genotype showed a marked decrease in total phenol content, particularly rutin. Total flavonoids were also the most abundant polyphenols in our study, except for the treatments CM_0_ (control treatment), CM_111_ (100:100:100 mg/mL N:P:K), CM_311_ (300:100:100 mg/mL N:P:K), and CM_322_ (300:200:200 mg/mL N:P:K) of the 2nd harvest, where phenolic acids, especially trans-*O*-Caffeoylqunic acid, were detected in higher amounts. In contrast, Nabet et al. (63) detected ten hydroxycinnamic acids in the hydromethanolic extracts of *C. maritimum* aerial parts, including those identified in our study, whereas no flavonoids were identified. Similarly, Piatti et al. (64) suggested that hydroxycinnamic acids were the prevalent polyphenols in aqueous and hydroethanolic extracts of *C. maritimum* aerial parts, especially caffeoylquinic acid derivatives, which were detected in higher amounts, while the same authors highlighted the impact of the protocol on the extraction yield of polyphenols, with hydroethanolic extracts at 60 °C showing the highest extraction yield. The same trend was observed by Alemán et al. (65), who also noted that large molecules of flavonoids could only be detected in hydroethanolic extracts, while chlorogenic acid was the most abundant compound in both hydroethanolic and aqueous extracts. Finally, Jallali et al. (66), who analysed the hydromethanolic extracts of *C. maritimum* leaves, identified 16 compounds in total, including eight phenolic acids, four flavonoids, and four flavanols, while the major compound was chlorogenic acid and its derivatives (neochlorogenic and cryptochlorogenic acid), followed by ferulic acid, kaempferol, and quercetin. Moreover, the total phenol content reported by Martins-Noguerol et al. (1) for the cultivated ecotype was within the range of our study, although the authors suggested that wild ecotypes had a notably higher total phenol content than the cultivated one. This finding highlights the impact that agronomic practices may have on polyphenol biosynthesis and the accumulation of secondary metabolites (1). In addition to agronomic practices, environmental conditions and genotype also play a crucial role in the configuration of the polyphenol profile, as Generalić Mekinić et al. (57) noted remarkable differences in phenolic acid and flavonoid content among ten Croatian *C. maritimum* ecotypes. Finally, the composition of polyphenols could be differentiated depending on the growth stage and phenology of plants at harvest, with flavonoid content being higher in the vegetative stage than during the flowering period, whereas the opposite trend was recorded for phenolic acids.

### 3.6 Antioxidant activity

The antioxidant activity of *C. maritimum* hydroethanolic extracts was determined with two cell-based assays, namely, TBARS and OxHLIA (Table 7). The IC_50_ values for both assays varied depending on the fertilization regime and harvest. In particular, the highest activity for TBARS assay was recorded for the CM_333_ (300:300:300 mg/mL N:P:K) and CM_111_ (100:100:100 mg/mL N:P:K) in the 1st and 2nd harvest, respectively, while the extracts of CM_0_ (control treatment), CM_222_ (200:200:200 mg/mL N:P:K), and CM_333_ (300:300:300 mg/mL N:P:K) recorded the lowest IC_50_ values in the 3rd harvest. Interestingly, the lowest activity was noted for CM_211_ (200:100:100 mg/mL N:P:K), regardless of the harvest. For the OxHLIA assay, CM_211_ (200:100:100 mg/mL N:P:K) and CM_333_ (300:300:300 mg/mL N:P:K) had the highest activity in the 1st harvest, while CM_333_ and CM_322_ showed the best performance in the 2nd and 3rd harvests, respectively. Moreover, the lowest activity was recorded for CM_311_ (300:100:100 mg/mL N:P:K) in the 1st harvest, CM_0_ (control treatment) and CM_111_ (100:100:100 mg/mL N:P:K) in the 2nd harvest, and CM_211_ (200:100:100 mg/mL N:P:K) in the 3rd harvest. Finally, the lowest overall activity for the OxHLIA assay was recorded in the 2nd harvest for all the fertilization regimes, while the same trend was also observed for the TBARS assay, except for treatments CM_211_ (200:100:100 mg/mL N:P:K), where no differences were recorded between the 2nd and 3rd harvests, and CM_333_ (300:300:300 mg/mL N:P:K), where the 1st harvest showed the lowest activity.

**Table 7:** Antioxidant properties (IC_50_ [μg/mL] values) of *Crithmum maritimum* aerial parts (mean ± SD; *n* = 3).

	TBARS	OxHLIA, Δt = 60 min
*1st Harvest*		
CM_0_	365 ± 13^d^	314 ± 15^b^
CM_111_	265 ± 4^e^	317 ± 15^b^
CM_211_	623 ± 10^a^	145 ± 8^c^
CM_222_	239 ± 2^f^	160 ± 9^c^
CM_311_	481 ± 7^b^	354 ± 20^a^
CM_322_	382 ± 4^c^	126 ± 7^d^
CM_333_	109 ± 2^g^	147 ± 9^c^
*2nd Harvest*		
CM_0_	136 ± 3^d^	142 ± 7^a^
CM_111_	55 ± 1^g^	144 ± 8^a^
CM_211_	353 ± 5^a^	48 ± 3^e^
CM_222_	89 ± 1^f^	130 ± 5^b^
CM_311_	177 ± 10^b^	114 ± 7^c^
CM_322_	150 ± 4^c^	77 ± 4^d^
CM_333_	125 ± 3^e^	18 ± 1^f^
*3rd Harvest*		
CM_0_	174 ± 8^d^	589 ± 36^b^
CM_111_	207 ± 2^c^	520 ± 16^c^
CM_211_	348 ± 10^a^	760 ± 28^a^
CM_222_	174 ± 5^d^	206 ± 11^d^
CM_311_	306 ± 3^b^	145 ± 8^f^
CM_322_	211 ± 4^c^	108 ± 6^g^
CM_333_	171 ± 7^d^	176 ± 9^e^
Mean values (range of variability) of the antioxidant properties		
CM_0_	225.3 (136–365)	348.3 (142–589)
CM_111_	175.3 (55–265)	327.0 (144–520)
CM_211_	435.7 (348–623)	317.7 (48–760)
CM_222_	167.3 (89–239)	165.3 (130–206)
CM_311_	321.0 (177–481)	204.3 (114–354)
CM_322_	246.0 (150–382)	103.7 (77–126)
CM_333_	133.3 (109–171)	113.7 (18–176)

In each harvest, values within the same column that are labelled with different lowercase letters differ significantly (*p* < 0.05), as determined by Tukey’s honest significance difference (HSD) test. IC_50_ values of Trolox: 139 ± 5 (TBARS) and 21.8 ± 0.2 (OxHLIA).

Several studies have reported the significant antiradical activities of extracts obtained from *C. maritimum* plant parts and essential or seed oils, highlighting the presence of a broad spectrum of functional compounds. However, the published results show a great variation due to differences in the implemented assays and extraction protocols, as well as in differences in the studied material due to variable genotypic background, environmental conditions, and agronomic conditions. Therefore, the review article by Kraouia et al. (9) indicates that antioxidant activity has been reported in hydromethanolic and hydroethanolic extracts using DPPH (2,2-Diphenyl-1-picrylhydrazyl), ABTS (2,2-azino-bis-3- ethylbenzothiazoline-6-sulfonic acid), ORAC (Oxygen Radical Absorbance Capacity), and FRAP (Ferric Reducing Antioxidant Power). The antiradical effects were associated with the high content of chlorogenic acid (46, 67), whereas Jallali et al. (66) suggested that other polyphenols may also contribute to the overall antioxidant capacity of *C. maritimum* depending on the extraction protocol. The varied scavenging activity of radicals of *C. maritimum* was also reported by Pereira et al. (16), who detected significant differences between two extraction protocols (e.g., decoctions and infusions) for leaves, stems, and flowers for eight antioxidant activity assays (five radical-based and three metal chelation methods). The genotypic effect is also important since ecotypes of *C. maritimum* may differ in the content of secondary metabolites that contribute to the overall defence mechanisms of plants and their antioxidant capacity (57, 68). Regarding the effect of fertilization on antioxidant activity, it has been suggested that the increase of the applied rates up to an optimal point is associated with increased antioxidant activity in pumpkin, while further increases are followed by decreased activity (69). In contrast, Oloyede et al. (70) reported that the highest antioxidant activity in pumpkins was observed in untreated plants or those that received low nutrient inputs owing to decreases in phenolic compound content. In the same line, Borgognone et al. (71) suggested that nitrate deprivation significantly increased antioxidant activity in cardoon leaves, as nitrogen availability is associated with inhibition of phenolic compounds biosynthesis and consequently with decreased antioxidant activity. However, the results of our study do not support a direct connection of phenolic compounds accumulation and antioxidant activity. A partial correlation was observed for OxHLIA activity for the 3rd harvest, where the high content of tocopherols and flavonoids was followed by the highest activity for this particular assay. Therefore, it could be suggested that other compounds not measured in this work could also contribute to the overall activity of *C. maritimum* leaf extracts, since the literature data show contrasting reports depending on the species, the nutrient rate, and the plant part (72).

### 3.7 Antimicrobial properties and anti-inflammatory activity

Tables 8, 9 present the antimicrobial activities of the hydromethanolic extracts of *C. maritimum* leaves against foodborne pathogens (Table 8) and clinical bacteria (Table 9), as well as their anti-inflammatory effects in relation to fertilization regime and harvesting time (Table 9). Our results indicate that none of the studied samples showed significant antimicrobial efficacy against any of the tested bacteria and fungi compared to streptomycin, which was used as a positive control. However, a variable response was recorded among the samples depending on the bacterial and fungal strain, fertilization rate, and harvesting time. For the foodborne pathogens, the control treatment (CM_0_, no fertilizers added) showed moderate efficacy (MIC values between 2.5 and 5 mg/mL) against *Escherichia coli*, *Yersinia enterocolitica*, *Bacillus cereus*, and *Staphylococcus aureus* in all harvests, while most treatments were efficient against *Salmonella enterica* in the 1st harvest, and against *Staphylococcus aureus* in the 1st and 3rd harvests (Table 8). Moreover, all treatments, except for the control treatment, were moderately efficient against *Listeria monocytogenes*, for example, CM_322_ (300:200:200 mg/mL N:P:K) and CM_333_ (300:300:300 mg/mL N:P:K) in the 1st harvest, CM_111_ (100:100:100 mg/mL N:P:K) and CM_211_ (200:100:100 mg/mL N:P:K) in the 2nd harvest, and CM_111_ (100:100:100 mg/mL N:P:K), CM_211_ (200:100:100 mg/mL N:P:K), and CM_311_ (300:100:100 mg/mL N:P:K) in the 3rd harvest. None of the studied extracts showed inhibitory activity against the bacterial strains of *Enterobacter cloacae* and *Pseudomonas aeruginosa* or against the fungi *Aspergillus brasiliensis* and *A. fumigatus*.

**Table 8:** Minimum inhibitory concentration (mg/mL) of the hydroethanolic extracts of *Crithmum maritimum* aerial parts against bacteria (ATCC strains) and fungi related to food isolates.

	Bacteria								Fungi	
	*Enterobacter cloacae*	*Escherichia coli*	*Pseudomonas aeruginosa*	*Salmonella enterica*	*Yersinia enterocolitica*	*Bacillus cereus*	*Listeria monocytogenes*	*Staphylococcus aureus*	*Aspergillus brasiliensis*	*Aspergillus fumigatus*
*1st Harvest*										
CM_0_	>10	5	10	10	5	5	10	5	>10	10
CM_111_	10	10	10	5	10	10	10	5	>10	5
CM_211_	10	10	10	5	10	10	10	5	>10	10
CM_222_	10	10	10	5	10	10	10	5	>10	10
CM_311_	10	10	10	5	10	5	5	5	>10	10
CM_322_	10	10	10	10	10	10	2.5	10	>10	10
CM_333_	10	10	10	10	10	10	2.5	10	>10	10
*2nd Harvest*										
CM_0_	10	5	10	10	2.5	5	10	2.5	>10	10
CM_111_	10	10	10	2.5	10	10	2.5	5	>10	10
CM_211_	10	10	10	10	10	2.5	2.5	5	>10	10
CM_222_	>10	10	>10	10	10	10	5	10	>10	10
CM_311_	>10	10	>10	10	10	10	5	10	>10	10
CM_322_	>10	10	>10	10	10	5	10	10	>10	10
CM_333_	>10	10	>10	10	5	10	10	10	>10	10
*3rd Harvest*										
CM_0_	>10	5	10	10	5	5	10	5	>10	10
CM_111_	>10	10	10	5	10	10	2.5	5	>10	10
CM_211_	>10	10	10	10	10	5	2.5	5	>10	10
CM_222_	>10	10	10	5	10	10	5	10	>10	10
CM_311_	>10	10	10	10	10	10	2.5	5	>10	10
CM_322_	>10	10	10	10	10	2.5	10	10	>10	10
CM_333_	>10	10	>10	10	5	10	10	10	>10	10

Minimum inhibitory concentration (MIC, mg/mL) of Streptomycin (1 μg/μL) was 0.007 for *E. cloacae*, *S. enterica*, *Y. enterocolitica*, *B. cereus*, *L. monocytogenes*, *S. aureus*, 0.01 for *E. coli*, and 0.06 for *P. aeruginosa*. Ampicillin (10 μg/μL) showed MIC values of 0.15 for *E. cloacae*, *E. coli*, *S. enterica*, *Y. enterocolitica*, *L. monocytogenes*, and *S. aureus*, and 0.63 for *P. aeruginosa*. Ketoconazole (1 μg/μL) exhibited MIC values of 0.06 for *A. brasiliensis* and 0.5 for *A. fumigatus*.

**Table 9:** Minimum inhibitory concentration (mg/mL) against clinical bacteria and anti-inflammatory effect (μg/mL) of the hydroethanolic extracts of *Crithmum maritimum* aerial parts.

	Clinical bacteria								Anti-inflammatory effect
	*Escherichia coli*	*Klebsiella pneumoniae*	*Morganella morganii*	*Proteus mirabilis*	*Pseudomonas aeruginosa*	*Enterococcus faecalis*	*Listeria monocytogenes*	MRSA	RAW 264.7
*1st Harvest*									
CM_0_	10	10	10	10	>10	10	5	0.6	>400
CM_111_	10	10	10	10	10	5	10	5	>400
CM_211_	10	10	5	10	10	5	10	5	>400
CM_222_	10	10	5	10	10	5	10	5	>400
CM_311_	10	10	10	10	10	10	10	5	>400
CM_322_	10	10	10	10	10	2.5	10	2.5	>400
CM_333_	10	10	10	10	10	2.5	10	1.25	>400
*2nd Harvest*									
CM_0_	10	10	10	>10	>10	10	5	1.25	>400
CM_111_	10	10	10	10	10	1.25	10	1.25	>400
CM_211_	10	10	10	10	10	2.5	10	5	>400
CM_222_	10	>10	10	10	>10	5	5	2.5	>400
CM_311_	10	>10	5	10	>10	1.25	5	1.25	>400
CM_322_	10	>10	10	10	>10	2.5	5	1.25	>400
CM_333_	10	>10	10	10	>10	10	10	1.25	>400
*3rd Harvest*									
CM_0_	10	10	10	>10	>10	10	5	1.25	>400
CM_111_	10	10	10	10	10	5	10	2.5	>400
CM_211_	10	10	10	10	10	2.5	5	5	>400
CM_222_	10	>10	10	10	10	5	5	2.5	>400
CM_311_	10	10	10	10	10	2.5	5	2.5	>400
CM_322_	10	10	10	10	>10	2.5	5	5	>400
CM_333_	10	>10	10	10	>10	2.5	10	1.25	>400

MIC values (mg/mL) of Streptomycin (1 μg/μL) was <0.15 *E. coli*, *P. mirabilis*, *E. faecalis*, *L. monocytogenes* and MRSA, and 10 for *K. pneumoniae*. Methicillin (1 μg/μL) exhibited MIC values of <0.0078 for *E. coli*, *K. pneumoniae*, *M. morganii*, *P. mirabilis* and *E. faecalis*, and 0.5 for *P. aeruginosa*.

For the clinical bacteria, moderate inhibitory activity was recorded against *Enterococcus faecalis*, *Listeria monocytogenes,* and methicillin-resistant *Staphylococcus aureus* (MRSA) for most fertilization treatments across the harvests, while specific treatments showed moderate inhibitory activity against *Morganella morganii* (CM_211_ (200:100:100 mg/mL N:P:K) and CM_222_ (200:200:200 mg/mL N:P:K) in the 1st harvest and CM_211_ (200:100:100 mg/mL N:P:K) in the 2nd harvest) (Table 9). Finally, none of the studied extracts showed significant anti-inflammatory activity against RAW 264.7 macrophage cell lines (Table 9).

Several studies have reported the antimicrobial properties of *C. maritimum* essential oils; however, there is scarce literature on the effects of extracts obtained from the aerial parts of the plant. Meot-Duros et al. (73) tested the inhibitory activity of chloroformic, methanolic, and aqueous extracts (5:12:3) of *C. maritimum* leaves against 12 bacterial strains and yeasts and suggested a varied efficacy depending on the polarity of the extract fractions and bacterial strain. In particular, polar fractions were effective against *Salmonella arizonae*, *Pseudomonas aeruginosa*, and *P. fluorescens*, whereas apolar fractions inhibited *Micrococcus luteus* and *Salmonella arizonae* (73). According to literature reports, the bioactive properties of a plant extract may vary depending on the solvent used for the extraction, the extraction protocol conditions and the extraction yield of bioactive compounds, as well as any possible synergistic effects among the extracted compounds against targeted microorganisms (74–76). In contrast to our study, none of the fractions inhibited *S. aureus* and *L. monocytogenes*, and apolar fractions showed moderate efficacy against *B. cereus*. Moreover, Jallali et al. (68) suggested a genotypic variability in the antimicrobial properties of acetonic extracts of *C. maritimum* aerial parts against one Gram negative and one Gram positive bacterial strain (e.g., *E. coli* and *S. aureus*, respectively), whereas no activity was recorded against *P. aeruginosa* (Gram^+^) and *B. cereus* (Gram^−^), a result which is partially aligned with our study (we recorded a moderate inhibitory effect against *B. cereus* for some of the studied extracts). Similar results were also reported by Souid et al. (8), who recorded a dose-dependent efficacy of *C. maritimum* hydroethanolic extracts against *E. coli* and *S. aureus*, while they further suggested that the extracts were capable of inhibiting the biofilm formation of *S. aureus* and ascribed the antimicrobial effects to the presence of specific phenolic compounds. Another factor that affects the antimicrobial properties of the species is the plant part from which the extracts are obtained. Houta et al. (77) indicated a varied efficacy of the methanolic extracts of leaves, stems, flowers, and seeds against six bacterial strains, including *S. aureus*, *E. coli*, and *P. aeruginosa*, also tested in our study. In particular, seed extracts were the most effective against *S. aureus*, stems and seeds showed the highest inhibitory activity against *E. coli*, and stems recorded the highest inhibitory zone against *P. aeruginosa*. Therefore, the extraction protocol and the polarity of the selected solvent (78, 79), the chemical profile of the extracts (80), or the lack of synergistic interactions among the detected bioactive compounds may affect the antimicrobial and anti-inflammatory effects of natural matrices (81).

### 3.8 Correlation and cluster analysis

The correlation coefficients and probabilities of the studied variables are presented in the Supplementary Material (Supplementary Tables S1, S2, Supplementary Figure S2). Interestingly, antioxidant activity assays showed a weak correlation with *α*-tocopherol content, whereas moderate positive or negative correlations were recorded with the various fatty acids and fatty acid classes detected (coefficient values 0.5 < r < 0.6 or − 0.5 < r < − 0.6, Moreover, a weak positive and negative correlation with trehalose and sucrose was recorded for the TBARS and OxHLIA assays, respectively, while a weak negative correlation of TBARS with specific phenolic compound content was observed (e.g., peaks 1, 6, 7, and 12). The cluster analysis of all chemical components of the studied samples identified three distinct clusters based on their similarities in macronutrient, free sugars, organic acids, fatty acids, tocopherols, and phenolic compounds content, and the antioxidant activity of the hydroethanolic extracts (Supplementary Material, Figure S3). Each cluster comprised all fertilization treatments for each harvest.

## 4 Conclusion

Domesticating wild edible plants through commercial cultivation is key to introducing new, resilient crops to small-scale Mediterranean farms. High nutrient inputs significantly enhanced the crop performance and nutritional value of *C. maritimum* by increasing fat, protein, and sugar content. Although fertilization strategies can be tailored to optimize the content of specific bioactive compounds, such as α-tocopherol, the plant’s overall antioxidant and antimicrobial performance remained variable and moderate. Ultimately, while the extracts showed no significant anti-inflammatory effect, their selective moderate inhibitory activity against certain pathogens (e.g., *B. cereus*, *L. monocytogenes, E. faecalis* and MRSA) suggests potential for targeted functional applications. Therefore, it can be suggested that tailored nutrient solutions are a viable agronomic practice for the commercial exploitation of *C. maritimum* through increased fresh biomass yield and quality of edible leaves.

## Data Availability

The original contributions presented in the study are included in the article/Supplementary material, further inquiries can be directed to the corresponding authors.
