# Supplementary material for: Biochemical profile and biofunctional effects of Crithmum maritimum L. from three harvests under different N:P:K ratios
Source: Front Nutr. 2026 May 13;13:1833407. doi: 10.3389/fnut.2026.1833407 (PMC13212070; doi:10.3389/fnut.2026.1833407)
Supplement: Supplementary file 1 [file Table_1.DOCX]

**Table S1.** Meteorological data of indoors conditions ((Mean temperature (°C), Mean maximum temperature (°C), and Mean minimum temperature (°C)).

| Months | Mean temperature (°C) | Mean maximum temperature (°C) | Mean minimum temperature (°C) | Mean relative humidity (%) |
| --- | --- | --- | --- | --- |
|  | 2020 | | | |
| September | 23.7 | 41.7 | 8.5 | 63.0 |
| October | 17.5 | 42.7 | 6.1 | 77.7 |
| November | 14.4 | 39.7 | 2.4 | 82.6 |
|  | 2021 | | | |
| December | 10.8 | 33.8 | -3.6 | 81.2 |
| January | 9.7 | 33.4 | -4.5 | 81.9 |
| February | 11.0 | 30.4 | -0.6 | 64.7 |
| March | 15.2 | 34.0 | 3.6 | 61.2 |

**Table S2.** Meteorological data of outdoors conditions ((Mean temperature (°C), Mean maximum temperature (°C), Mean minimum temperature (°C), Mean relative humidity (%) and Rainfall (mm)) throughout the growing period.

| Months | Mean temperature (°C) | Mean maximum temperature (°C) | Mean minimum temperature (°C) | Mean relative humidity (%) | Rainfall (mm) |
| --- | --- | --- | --- | --- | --- |
|  | 2020 | | | | |
| April | 13.56 | 20.16 | 7.08 | 52.63 | 21.90 |
| May | 20.95 | 28.52 | 12.91 | 45.81 | 21.50 |
| June | 24.34 | 31.85 | 16.78 | 54.27 | 12.60 |
| July | 28.45 | 35.82 | 20.37 | 44.42 | 2.20 |
| August | 28.13 | 36.39 | 20.35 | 47.55 | 8.60 |
| September | 22.10 | 29.20 | 15.80 | 55.87 | 52.50 |
| October | 14.63 | 20.06 | 10.25 | 69.65 | 58.50 |
| November | 12.05 | 17.02 | 7.67 | 72.37 | 46.40 |
| December | 7.75 | 12.76 | 3.00 | 57.45 | 31.60 |
|  | 2022 | | | | |
| January | 4.65 | 11.21 | -0.54 | 52.71 | 84.70 |
| February | 7.81 | 14.29 | 2.34 | 57.46 | 69.10 |
| March | 7.19 | 13.00 | 1.55 | 49.13 | 41.00 |
| April | 14.92 | 21.94 | 7.94 | 49.37 | 21.60 |
| May | 20.27 | 27.81 | 12.46 | 50.10 | 16.30 |
| June | 25.60 | 32.42 | 18.45 | 53.13 | 62.50 |
| July | 26.81 | 33.65 | 19.08 | 44.84 | 17.00 |
| August | 26.44 | 33.20 | 19.77 | 52.58 | 48.40 |
| September | 22.54 | 29.74 | 15.43 | 53.23 | 20.30 |
| October | 16.71 | 24.28 | 10.42 | 57.81 | 19.00 |
| November | 13.77 | 19.41 | 8.90 | 65.67 | 60.50 |
| December | 9.40 | 13.72 | 5.91 | 77.32 | 20.90 |
|  | 2023 | | | | |
| January | 7.42 | 13.04 | 2.67 | 64.19 | 30.30 |
| February | 6.93 | 14.03 | 0.59 | 51.30 | 9.10 |
| March | 11.40 | 18.33 | 4.55 | 55.45 | 34.50 |
| April | 13.88 | 20.55 | 7.56 | 56.07 | 83.50 |
| May | 17.63 | 23.56 | 11.92 | 61.39 | 27.10 |
| June | 23.28 | 30.09 | 15.97 | 53.87 | 22.60 |
| July | 29.47 | 36.96 | 20.77 | 41.68 | 0.00 |
