# Supplementary material for: Biochemical profile and biofunctional effects of Crithmum maritimum L. from three harvests under different N:P:K ratios
Source: Front Nutr. 2026 May 13;13:1833407. doi: 10.3389/fnut.2026.1833407 (PMC13212070; doi:10.3389/fnut.2026.1833407)
Supplement: Supplementary file 2 [file Table_2.DOCX]

**Figure S1.** Chromatographic profile of phenolic coumponds obtained from hydroethanolic extracts of *C. maritimum* leaves and stems recorded at 280 nm (a) and 330 nm (b) in HPLC.


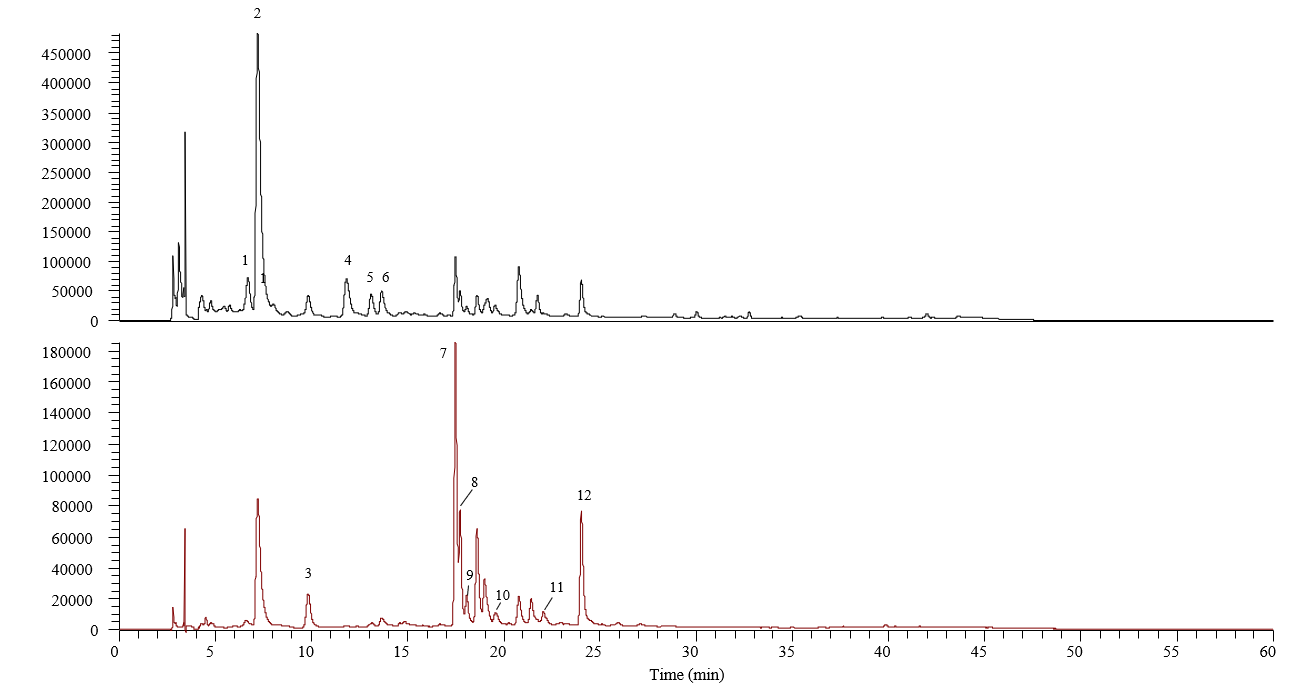


(a)

(b)


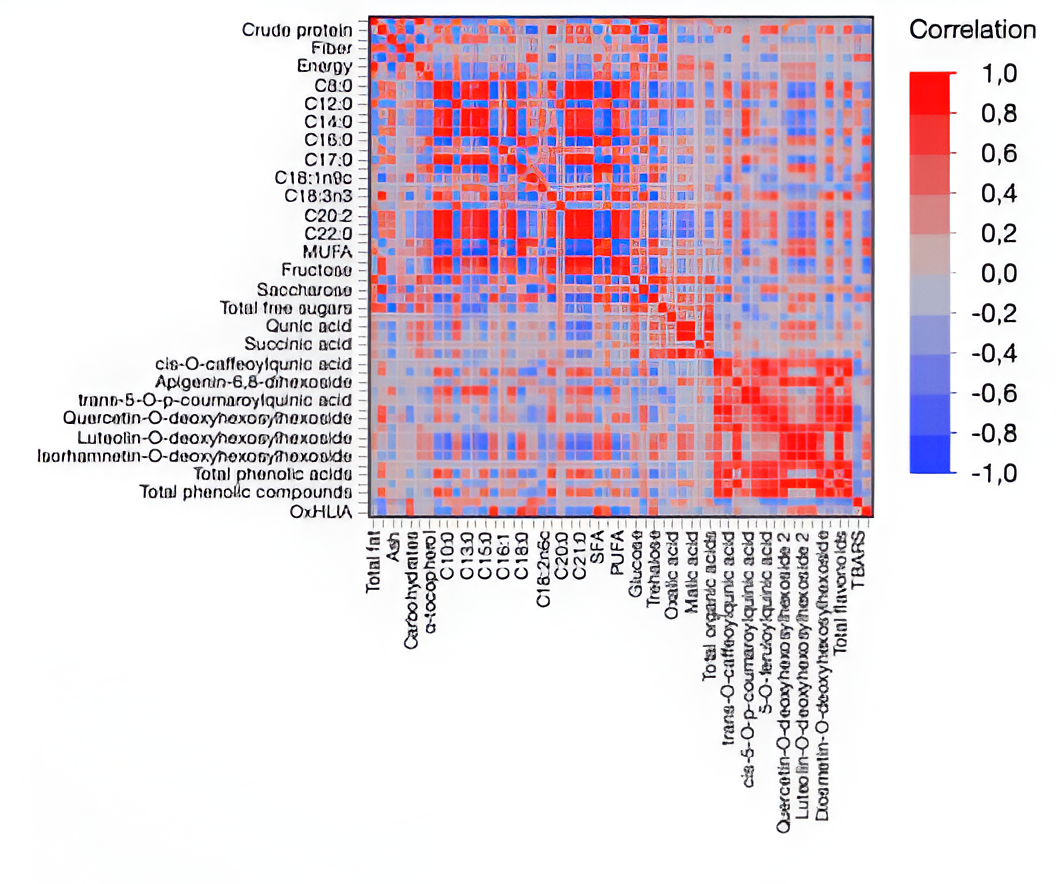


**Figure S2**. Correlation heatmap of chemical components of *Crithmum maritimum* in relation to fertilization regime and harvesting time.


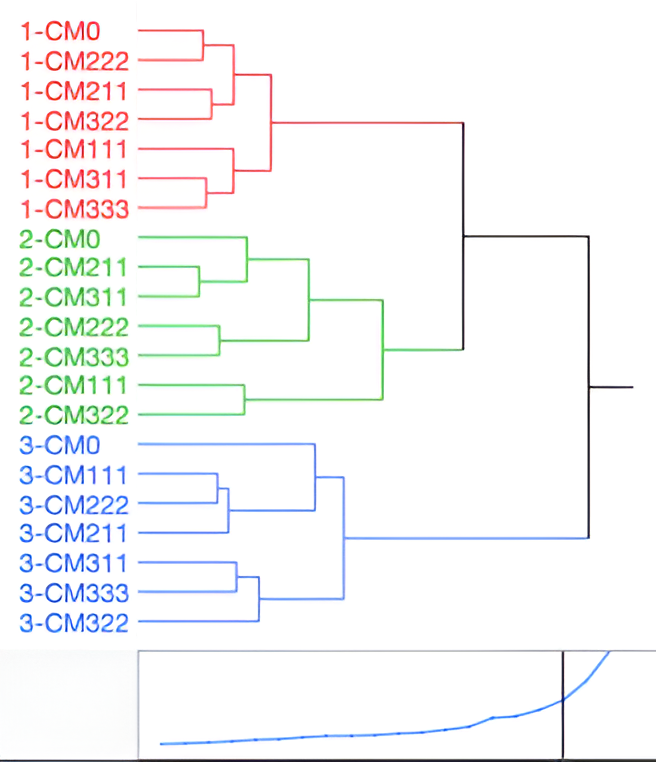


**Figure S3**. The dendrogram of the hierarchical cluster analysis of chemical components of *Crithmum maritimum* in relation to fertilization regime and harvesting time. Individual clusters are indicated by different colors
